# Efficacy of haloperidol to decrease the burden of delirium in adult critically ill patients: the EuRIDICE randomized clinical trial

**DOI:** 10.1186/s13054-023-04692-3

**Published:** 2023-10-30

**Authors:** Lisa Smit, Arjen J. C. Slooter, John W. Devlin, Zoran Trogrlic, Nicole G. M. Hunfeld, Robert Jan Osse, Huibert H. Ponssen, Arjen J. B. W. Brouwers, Jeannette F. Schoonderbeek, Koen S. Simons, Mark van den Boogaard, Judith A. Lens, Dirk P. Boer, Diederik A. M. P. J. Gommers, Wim J. R. Rietdijk, Mathieu van der Jagt, the EuRIDICE study group

**Affiliations:** 1 Department of Intensive Care Adults, Erasmus MC-University Medical Centre, Room Ne-415, PO BOX 2040, 3000 CA Rotterdam, The Netherlands; 2 Departments of Psychiatry, Intensive Care Medicine and UMC Utrecht Brain Center, University Medical Center Utrecht, Utrecht University, Utrecht, The Netherlands; 3 Department of Neurology, UZ Brussel and Vrije Universiteit Brussel, Brussels, Belgium; 4 School of Pharmacy, Northeastern University, Boston, USA; 5 Division of Pulmonary and Critical Care Medicine, Brigham and Women’s Hospital, Boston, USA; 6 Department of Hospital Pharmacy, Erasmus MC-University Medical Centre, Rotterdam, The Netherlands; 7 Department of Psychiatry, Erasmus MC-University Medical Centre, Rotterdam, The Netherlands; 8 Department of Intensive Care, Albert Schweitzer Hospital, Dordrecht, The Netherlands; 9 Department of Intensive Care Adults, Franciscus Gasthuis & Vlietland, Rotterdam, The Netherlands; 10 Department of Intensive Care, Ikazia Hospital, Rotterdam, The Netherlands; 11 Department of Intensive Care Medicine, Jeroen Bosch Hospital, ‘s Hertogenbosch, The Netherlands; 12 Department of Intensive Care Medicine, Radboud University Medical Center, Nijmegen, The Netherlands; 13 Department of Intensive Care, IJsselland Hospital, Capelle aan den IJssel, The Netherlands; 14 Department of Intensive Care, Maasstad Hospital, Rotterdam, The Netherlands

**Keywords:** Delirium, Haloperidol, Randomized controlled trial, Antipsychotic, Critical care

## Abstract

**Background:**

The role of haloperidol as treatment for ICU delirium and related symptoms remains controversial despite two recent large controlled trials evaluating its efficacy and safety. We sought to determine whether haloperidol when compared to placebo in critically ill adults with delirium reduces days with delirium and coma and improves delirium-related sequelae.

**Methods:**

This multi-center double-blind, placebo-controlled randomized trial at eight mixed medical-surgical Dutch ICUs included critically ill adults with delirium (Intensive Care Delirium Screening Checklist ≥ 4 or a positive Confusion Assessment Method for the ICU) admitted between February 2018 and January 2020. Patients were randomized to intravenous haloperidol 2.5 mg or placebo every 8 h, titrated up to 5 mg every 8 h if delirium persisted until ICU discharge or up to 14 days. The primary outcome was ICU delirium- and coma-free days (DCFDs) within 14 days after randomization. Predefined secondary outcomes included the protocolized use of sedatives for agitation and related behaviors, patient-initiated extubation and invasive device removal, adverse drug associated events, mechanical ventilation, ICU length of stay, 28-day mortality, and long-term outcomes up to 1-year after randomization.

**Results:**

The trial was terminated prematurely for primary endpoint futility on DSMB advice after enrolment of 132 (65 haloperidol; 67 placebo) patients [mean age 64 (15) years, APACHE IV score 73.1 (33.9), male 68%]. Haloperidol did not increase DCFDs (adjusted RR 0.98 [95% CI 0.73–1.31], *p* = 0.87). Patients treated with haloperidol (vs. placebo) were less likely to receive benzodiazepines (adjusted OR 0.41 [95% CI 0.18–0.89], *p* = 0.02). Effect measures of other secondary outcomes related to agitation (use of open label haloperidol [OR 0.43 (95% CI 0.12–1.56)] and other antipsychotics [OR 0.63 (95% CI 0.29–1.32)], self-extubation or invasive device removal [OR 0.70 (95% CI 0.22–2.18)]) appeared consistently more favorable with haloperidol, but the confidence interval also included harm. Adverse drug events were not different. Long-term secondary outcomes (e.g., ICU recall and quality of life) warrant further study.

**Conclusions:**

Haloperidol does not reduce delirium in critically ill delirious adults. However, it may reduce rescue medication requirements and agitation-related events in delirious ICU patients warranting further evaluation.

*Trial registration*: ClinicalTrials.gov (#NCT03628391), October 9, 2017.

**Supplementary Information:**

The online version contains supplementary material available at 10.1186/s13054-023-04692-3.

## Background

Delirium is an acute neuropsychiatric syndrome occurring in up to 50% of critically ill adults [1]. It is associated with longer hospital stay, long-term cognitive dysfunction and increased costs [2–4]. Haloperidol has been the preferred agent to treat delirium for decades [5]. Current guidelines make a conditional recommendation against the routine administration of haloperidol to treat delirium in critically ill adults, only considering its use in case of significant distress or agitation [6]. Instead, these guidelines advocate the use of non-pharmacologic strategies, including the ABCDEF bundle [6]. However, common delirium symptoms, including agitation, hallucinations and delusions, often respond poorly to non-pharmacologic treatments.

Current evidence regarding the use of haloperidol to treat ICU delirium is derived from two recent major trials (MIND-USA and AID-ICU trial [7, 8]) that found haloperidol to be ineffective in reducing delirium duration [9]. Importantly, neither trial rigorously evaluated key delirium-related endpoints including agitation-related sequelae nor the occurrence of psychotic symptoms, although administration of haloperidol is usually targeted at these clinical endpoints in routine practice and these outcomes are likely just as important from both a clinician and patient/family perspective [10].

We conducted a multi-center, double-blind, placebo-controlled, randomized clinical trial to assess the efficacy and safety of haloperidol as treatment for delirium and its associated symptoms and outcomes in critically ill adults.

## Methods

### Study design, setting and participants

We conducted a multi-center randomized, double-blind, placebo-controlled clinical trial in adults with delirium at eight ICUs in the Netherlands. The study protocol was approved by the Medical Ethics Committees of all participating hospitals and has been published [11]. An independent data and safety monitoring board (DSMB) provided oversight of the trial.

All adult (≥ 18 years) ICU patients were eligible unless they met one or more exclusion criteria: admission to the ICU because of a primary acute neurological condition; pregnancy or breast-feeding; known allergy to haloperidol; history of ventricular arrhythmia (including torsade de pointes); neuroleptic malignant syndrome; parkinsonism; schizophrenia or other psychotic disorder; dementia or an Informant Questionnaire on Cognitive Decline in the Elderly (IQCODE) score ≥ 4[12]; expected duration of ICU admission < 24 h; inability to speak the Dutch language or to undergo a valid delirium assessment (e.g., deafness or blindness); or participation in another interventional trial [11]. Eligible patients or their legal representatives were asked for written informed consent as soon as possible after ICU admission to enable randomization as soon as possible after delirium was first diagnosed.

Trained ICU nurses evaluated each patient three times daily (once per 8-h shift) for level of sedation with the Richmond Agitation Sedation Scale (RASS) [13] and for delirium with either the Intensive Care Delirium Screening Checklist (ICDSC) [14] or the Confusion Assessment Method for the ICU (CAM-ICU) [15] in the absence of coma (RASS score − 4 or − 5). Each participating ICU used one of these delirium assessment methods consistently and non-interchangeably [16]. Three ICUs used the ICDSC and five used the CAM-ICU. Once eligible patients with written informed consent developed delirium (a positive CAM-ICU or an ICDSC score ≥ 4), they were randomized if none of the above exclusion criteria were present, and none of the additional criteria for randomization were met: QTc prolongation (QTc > 500 ms), acute alcohol (or substance) withdrawal syndrome, an expected ICU stay < 24 h, or torsade de pointes, neuroleptic malignant syndrome or parkinsonism since ICU admission [11]. During the study, we encountered eligible patients that were missed for randomization and already treated with haloperidol at the ICU > 24 h or received more than 3 doses, and they were also excluded from randomization.

### Randomization and masking, study procedures and interventions

Randomization was computer-generated using a block design of eight patients per block (4 haloperidol and 4 placebo in random order), stratified by participating center [11]. The haloperidol and placebo ampoules were identical in appearance. Only the involved site-pharmacists and the trial statistician had access to the contents of each medication kit.

The study drug was administered intravenously, starting with 2.5 mg three times daily (for patients ≥ 80 years 1 mg), and increased up to 5 mg three times daily (for patients ≥ 80 years 2.5 mg) if delirium persisted in the next 8-h shift. The first dose was administered immediately after randomization or at the subsequent regular medication prescription in the electronic patient system (every 8 h). When delirium had resolved (or was not assessable due to coma) for 24 h, study drug was decreased (from 5 to 2.5 mg for patients < 80 years or from 2.5 to 1 mg for patients ≥ 80 years) or stopped (if at a dose of 2.5 mg for patients < 80 years or 1 mg for patients ≥ 80 years). The study drug was restarted if delirium re-occurred within the 14-day intervention period and the patient remained at the ICU. At the discretion of the ICU clinical team, and after consultation with the research team when necessary, doses could be lowered (or held) when safety concerns presumably related to haloperidol were suspected (i.e., drug associated adverse effects, as described in Additional file 1: S1). After the 14-day intervention period, treatment with haloperidol was permitted as per local treatment protocol.

Open-label haloperidol during the 14-day intervention period was strongly discouraged. If agitation did not resolve after potential causes (e.g., acute pain) were identified and treated, it could be treated with an alpha-2 agonist (e.g., dexmedetomidine, clonidine) or GABA agonist (e.g., propofol, benzodiazepine). Patients appearing to be in distress (e.g., from hallucinations) could be treated with a low-dose atypical antipsychotic. Standard clinical practice was followed according to clinical protocols, based on the clinical practice guidelines from the Society of Critical Care Medicine that were implemented in a previous multi-center implementation study [6, 16, 17]. At the start of the study, all centers were subjected to a qualitative survey on perceived adherence to components of the ABCDE (awakening and breathing coordination, choice of sedation, delirium monitoring and management, and early mobility) bundle (Additional file 1: S2). Quality ascertainment of delirium assessments with spot checks assured accurateness of > 90% (Additional file 1: S2).

### Outcomes

The primary outcome was the number of delirium- and coma-free days (DCFDs) while alive and admitted to the ICU up to 14 days after randomization. A delirium day was defined as at least one positive delirium assessment on that calendar day where the RASS score was ≥ − 3. A patient was considered to be comatose when any RASS score on that day was − 4 or − 5 in the absence of documented delirium or if delirium was indicated to be not assessable due to coma [7]. Patients who died within the intervention period were considered to have 0 DCFDs for the whole intervention period, in line with previous intervention trials [18, 19]. Patients who were discharged from the ICU during the intervention period were considered to be delirium- and coma-free after ICU discharge, regardless of their delirium status at discharge [7, 20]. We conducted prespecified subgroup analyses on motor subtype, presence of hallucinations or delusions, delirium severity, and delirium phenotype [11].

All outcomes refer to the 14-day intervention period, which started after randomization. Predefined secondary outcomes were daily RASS scores, maximum mobility level, sleep quality, use of “escape medication” for hallucinations and/or agitation (including atypical antipsychotics, alpha-2 agonists [clonidine and dexmedetomidine], GABA agonists [benzodiazepines and propofol], opiates and ‘open-label’ haloperidol), daily study drug dose corrected for body weight (mg/kg), self-extubation rate and patient-initiated removal of invasive devices, (serious) adverse drug associated events (prolonged QTc by EKG, muscle rigidity and other associated movement disorders [Simpson Angus Scale] [21], ventricular arrhythmias including torsade de pointes), blood pressure after first study drug dose, daily respiratory status, time from randomization to first resolution of delirium, time to readiness for discharge from the ICU and 28-day mortality [11].

We also assessed the following prospectively collected post hoc exploratory outcomes: number of days with study drug administration, and number of days with delirium, coma, agitation (defined as a RASS score + 2 or more at least once on that day[18]) or hallucinations/delusions, auto-removal of urinary catheter, physical restraint and (almost) fell or stepped out of bed.

Secondary predefined long-term outcomes after hospital discharge were patient and family member memories and experiences at hospital discharge and 3 months after randomization, post-traumatic stress disorder (PTSD) symptoms and burden experienced by the family at 3 months after randomization, anxiety, depression, cognition, and quality of life at 3 and 12 months after randomization, and 12-month mortality.

Data collection and definitions of all subgroup analyses and outcomes are described in detail in Additional file 1: S1 and in the EuRIDICE study protocol [11].

### Sample size calculation and statistical analyses

We aimed to include 742 patients (371 in each group) to provide 90% power to detect a one-day difference in DCFDs (alpha level of 0.05, with non-parametric testing) assuming an average prevalence of 3.2 DCFDs in the control group and 4.2 in the haloperidol group (standard deviation [SD] in both groups 4.2); the estimates were based on a previous study by our group [16].

Categorical data were presented as numbers (percentages) and continuous data as means (SD) or median (interquartile range [IQR]). Categorical data were compared using Chi-square tests or Fisher’s Exact Tests. For continuous data, independent sample t-tests or Mann Whitney-*U* tests were used, all when appropriate.

For the primary outcome, we analyzed differences between the groups with a Poisson or negative binomial mixed effects model, depending on the presence of overdispersion. We performed adjusted analyses based on significant differences in baseline characteristics between treatment groups (when present) as covariates and hospital as a random effect.

For the secondary outcomes, we adjusted analyses similarly. For data with a Poisson distribution, we used a Poisson or negative binomial mixed effects model. For other continuous secondary outcomes, linear mixed effects models were used. We applied a mixed effects Cox proportional hazards model for time to ICU discharge, censoring at death, withdrawal of consent before ICU discharge, and loss to follow-up (i.e., transferred to another ICU not participating in the trial). Additionally, categorical secondary outcomes were analyzed with logistic mixed effects models. Mortality risk was assessed as a binary endpoint at 28-days after randomization. One-year mortality was expressed as time until death, calculated as time from randomization until death, and was analyzed with a mixed-effect Cox proportional model using hospital as a random effect and censoring if death had not occurred during the 1-year follow-up, if informed consent was withdrawn or if patients were lost to follow-up during the 1-year follow-up. We analyzed all data using an intention-to-treat-principle.

We used SPSS (version 25) and R software (version 1.3.1073). A *p* value < 0.05 (two-sided) was considered statistically significant. The results are reported using the CONSORT statement [22].

### Interim analysis and trial termination

Interim analyses, focused on safety and futility, were pre-specified by a DSMB charter to occur at the one-third and two-third enrolment benchmarks or every 6 months, whichever occurred first. Recruitment started in February 2018. Due to slow recruitment, the steering committee and DSMB agreed to decrease the statistical power from 90 to 80% in April 2019, lowering the intended sample size to 554 patients. Based on a subsequent pre-planned interim analyses conducted on October 31, 2019 (*n* = 118), the DSMB advised to stop recruitment on December 19, 2019, because of expected futility of being able to reach a significant one-day difference between treatment groups in the primary outcome of DCFD in the intended sample size of 554 patients. Patient recruitment for the trial was terminated on January 22, 2020, at 26% of the intended sample size (*n* = 142).

## Results

### Patient characteristics and interventions

**Fig. 1:**
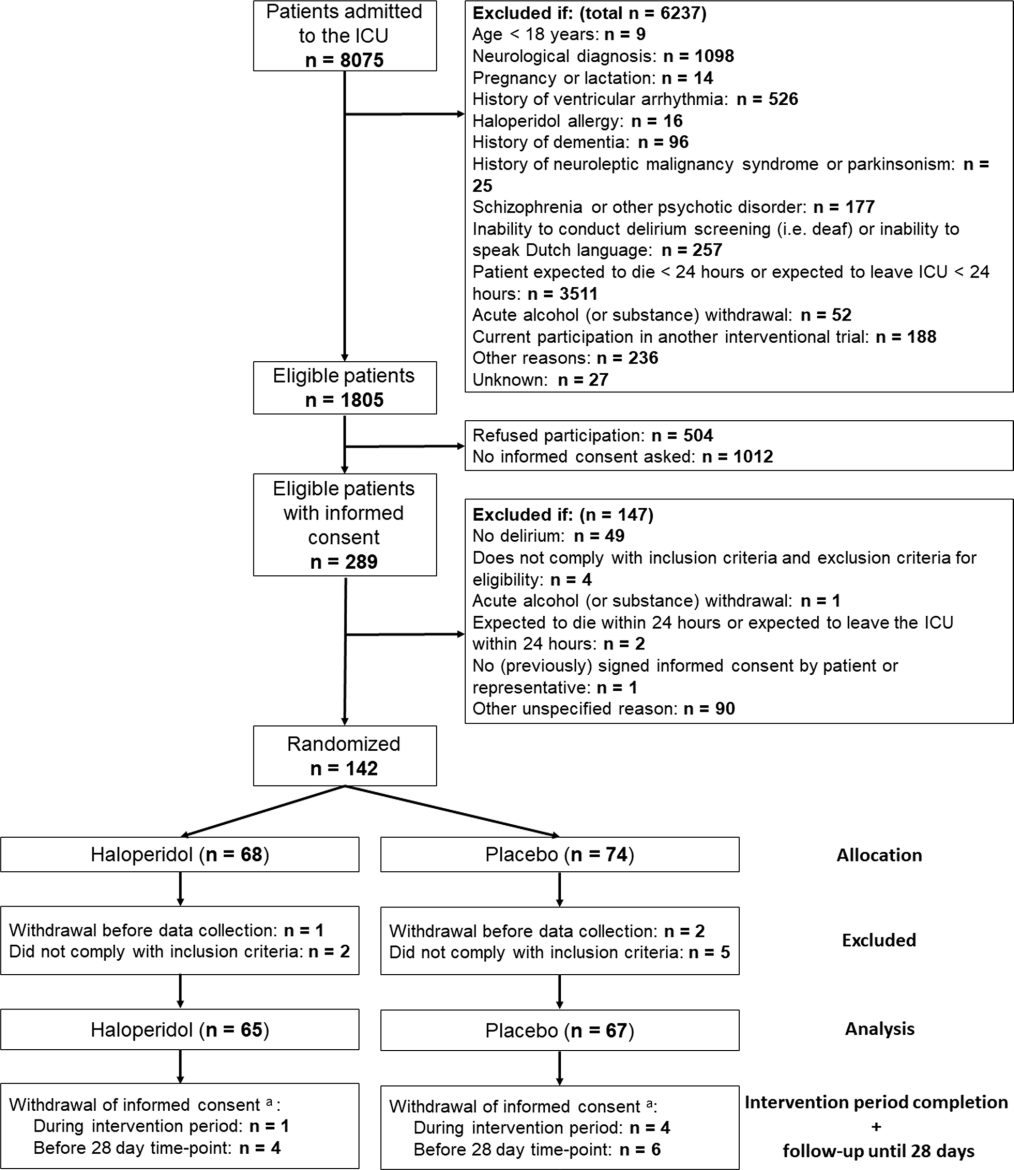
Flow of participants in the EuRIDICE trial. ^a^None of the patients who withdrew informed consent rejected the use of their yet collected data. They, however, refused further participation in the trial

From February 21, 2018 to January 22, 2020, we screened 8075 ICU patients. Follow-up was completed on April 12, 2021, 15 months after randomization was halted based on the DSMB advice of further futility. Written informed consent was obtained from 289 (16%) of 1805 eligible patients (Fig. 1). Subsequently, 142 patients developed delirium and were randomized. Ten randomized patients were withdrawn before first study drug administration, because they did not comply with the inclusion criteria or withdrew informed consent. Consequently, we analyzed data from 132 patients (65 haloperidol, 67 placebo). A total of 91 patients were eligible for the follow-up period (45 haloperidol, 46 placebo; details provided in Additional file 1: S3).

The mean age of included patients was 64 years (SD 15.3), and 90 (68%) were male. The overall mean APACHE IV score was 73.1 (SD 33.9). The groups were similar at baseline, except for the median modified SOFA score (mSOFA) at randomization, which was significantly lower in the haloperidol group (*p* = 0.03), Table 1 and Additional file 1: Table E1. Information related to delirium screening instrument used in included patients is shown in Additional file 1: Table E2.

**Table 1:** Baseline characteristics of the patients

Characteristic	Haloperidol (*n* = 65)	Placebo (*n* = 67)
Age, median (IQR), year	66 (57–75.5)	68 (60–74)
Male, *n* (%)	48 (74)	42 (63)
Location before ICU admission		
Hospital ward, *n* (%)	29 (45)	31 (46)
Operation room/recovery, *n* (%)	17 (26)	18 (27)
ED, *n* (%)	12 (19)	11 (16)
Other, *n* (%)	7 (11)	7 (10)
Type of ICU admission		
Medical, *n* (%)	36 (55)	39 (58)
Emergency surgery, *n* (%)	17 (26)	17 (25)
Elective surgery, *n* (%)	12 (19)	11 (16)
ICU admission diagnosis		
Respiratory, *n* (%)	24 (37)	28 (42)
Gastrointestinal, *n* (%)	14 (22)	10 (15)
Cardiovascular, *n* (%)	9 (14)	16 (24)
Transplant, *n* (%)	7 (11)	5 (8)
Trauma, *n* (%)	5 (8)	1 (2)
Other, *n* (%)	6 (9)	7 (10)
APACHE IV score, median (IQR)	70 (57–90.5)	77 (55–99)
mSOFA score at randomization, median (IQR)	5 (3–7)*	6 (4–9)
Baseline QTc at randomization, mean (SD)	428.9 (29.9)	421.3 (32.1)
No. of days from ICU admission to randomization, median (IQR)	4 (2–10.5)	6 (3–9)

*APACHE* acute physiology and chronic health evaluation, *ED* emergency department, *ICU* intensive care unit, *mSOFA* modified sequential organ failure assessment (without the central nervous system component), *SOFA* sequential organ failure assessment, *Yr* year

*Significantly differed from the placebo group (*p* < 0.05)

### Primary outcome: delirium- and coma-free days

The median number of DCFDs was not different between the haloperidol (9 [IQR 3–12]) and placebo group (9 [IQR 2–11]), *p* = 0.66 (Table 2). After adjusting for mSOFA at randomization and a random effect for hospital, the number of DCFDs remained similar (adjusted RR [aRR] 0.98 [95% CI 0.73–1.31], *p* = 0.87, Table 2 and Additional file 1: Figures E1 and E2).

**Table 2:** Comparison of delirium- and coma-free days and predefined secondary outcomes between the haloperidol and placebo group

Outcome	Haloperidol (*n* = 65)	Placebo (*n* = 67)	Adjusted difference (95%CI)	Adjusted relative risk (95% CI)^a^	*p* value
*Primary outcome: delirium- and coma-free days*					
No. of DCFDs, median (IQR)^b^	9 (3–12)	9 (2–11)		0.98 (0.73–1.31)	0.871
*Predefined secondary outcomes: clinical outcomes*					
Daily RASS score, median (IQR)	− 0.5 (− 0.9 to − 0.1)	− 0.3 (− 0.6 to − 0.1)	− 9.9% (− 55.1% to 80.6%)^c^		0.777
Maximum mobility level, median (IQR)^d^	4 (1.5–5)	3 (1.8–5)	− 0.03 (− 0.8 to 0.74)		0.938
Sleep quality as assessed by nurse, mean (SD)^d^	4.2 (1.3)	4.4 (1.2)	− 0.26 (− 0.7 to 0.18)		0.251
Sleep quality according to patient (RCSQ), mean (SD)^d^	4.1 (1.4)	4.4 (1.5)	− 0.27 (− 0.92 to 0.38)		0.416
Mechanical ventilation, *n* (%)	50 (77)	51 (76)		OR: 1.17 (0.51–2.71)	0.707
No. of days, median (IQR)	2 (1–7.5)	2 (1–7)		1.17 (0.77–1.78)	0.465
Time to first resolution of delirium in days, median (IQR)	2 (1–3)	2 (1–4)	− 16.6% (− 35.5% to 7.7%)^c^		0.168
Median time to ICU discharge alive, days (95% CI)^d,e^	18.1 (9.8–26.4)	15.5 (12.3–18.7)		HR: 0.69 (0.47–1.02)	0.061
28-day mortality, *n* (%)^d^	10 (16)	13 (21)		0.79 (0.31–2.01)	0.622
*Predefined secondary outcomes: medication-related outcomes*					
Daily study drug corrected for body weight (mg/kg), median (IQR)	0.08 (0.05–0.11)	0.10 (0.08–0.13)	− 0.01 (− 0.03 to 0.00)		0.101
Use of “escape medication”, *n* (%)	64 (99)	64 (96)		OR: 3.59 (0.43–75.62)	0.283
Open-label haloperidol, *n* (%)	4 (6)	9 (13)		OR: 0.43 (0.12–1.56)	0.201
Open-label haloperidol, mean 24 h dose in mg, mean (SD)^c^	3.6 (3.2)	2.6 (1.1)	1.32 (− 0.9 to 3.54)		0.295
Atypical antipsychotic, n (%)^f^	23 (35)	32 (48)		OR: 0.63 (0.29–1.32)	0.223
Clonidine, *n* (%)	26 (40)	34 (51)		OR: 0.61 (0.28–1.30)	0.198
Dexmedetomidine, *n* (%)	13 (20)	16 (24)		OR: 0.87 (0.31–2.46)	0.787
Benzodiazepine, *n* (%)	37 (57)	49 (73)		OR: 0.41 (0.18–0.89)	0.028
Propofol, *n* (%)	39 (60)	38 (57)		OR: 1.43 (0.67–3.07)	0.357
Opioid, *n* (%)	57 (88)	60 (90)		OR: 0.94 (0.31–2.88)	0.919
Other sedatives, *n* (%)	3 (5)	6 (9)		OR: 0.42 (0.08–1.79)	0.256
*Predefined secondary outcomes: safety outcomes*					
Self-extubation or removal of invasive devices, ever, *n* (%)	6 (9.2)	10 (14.9%)		OR: 0.70 (0.22–2.18)	0.539
QTc prolongation, *n* (%)	3 (5)	6 (9)		OR: 0.62 (0.12–2.71)	0.535
Muscle rigidity and associated movement disorders, n (%)	3 (5)	1 (2)		OR: 4.52 (0.53–97.33)	0.211
Ventricular arrhythmia, n (%)	4 (6)	1 (2)		OR: 5.21 (0.71–105.98)	0.153
Systolic blood pressure change after first study drug dose, median (IQR)	− 5 (− 21 to 9.25)	2 (− 4.5 to 10)	− 12.41 (− 19.63 to 5.18)		0.001
Diastolic blood pressure change after first study drug dose, median (IQR)	− 3 (− 9 to 1)	2 (− 2 to 6.5)	− 7.96 (− 11.78 to − 4.20)		< .001

*CI* confidence interval, *DCFD* delirium- and coma-free day, *IQR* interquartile range, *OR* odds ratio, *RR* relative risk

^a^RR unless mentioned otherwise. The placebo group was used as a reference. The RR may be interpreted as follows: the number of DCFDs in the haloperidol group is 0.98 times the number in the placebo group

^b^The mean number of DCFDs in the haloperidol group was 7.4 (SD 4.7) and in the placebo group 7.2 (SD 4.8). In order to increase comparability and to assess the impact of assumptions related to the definition of DCFD, post hoc analyses were performed (Additional file 1: Online Supplement 4)

^c^After log transformation due to non-normality and/or heteroscedasticity, and needs to be interpreted as adjusted percent change for the haloperidol vs. placebo group. For example, compared to placebo, haloperidol decreases the median time to first resolution of delirium in days by 16.6 percent (adjusted percent change for haloperidol vs. placebo is − 16.6%, not significant) if all other variables are kept constant

^d^Data were missing for some patients: maximum mobility 1 (0.8%), mean sleep quality as assessed by nurse 5 (3.9%), mean sleep quality according to patient 50 (37.9%), systolic blood pressure change after first study drug administration 15 (11.4%), diastolic blood pressure change after first study drug administration 15 (11.4%), time to ICU discharge 3 (2.3%), 28-day mortality 10 (7.6%). No missing data were present for the other outcomes, study drug or covariates (mSOFA and hospital)

^e^Unadjusted differences were estimated with the Kaplan–Meier method

^f^Olanzapine or quetiapine

### Predefined secondary outcomes

The results of the secondary outcomes are presented in Table 2 and Additional file 1: Table E3. Significantly fewer haloperidol-treated (vs. placebo) patients ever received a benzodiazepine (57% vs. 73%, adjusted OR [aOR] 0.41 [95%CI 0.18–0.89], *p *= 0.03; both continuous infusion and intermittent, Additional file 1: Table E4). The patients in the haloperidol group had significantly lower systolic and diastolic blood pressure after the first study drug dose than the placebo group (adjusted difference in systolic blood pressure change: − 12.41 [95% CI − 19.63 to 5.18], *p* = 0.001). No statistically significant differences in adverse drug associated events were observed. There was no statistically significant difference in duration of ventilation and 28-day mortality. Effect measures of secondary outcomes related to agitation (use of open label haloperidol [aOR 0.43 (95% CI 0.12–1.56)] and other antipsychotics [aOR 0.63 (95% CI 0.29–1.32)] and self-extubation or invasive device removal [aOR 0.70 (95% CI 0.22–2.18)]) appeared consistently more favorable with haloperidol treatment, although the confidence interval also included no effect. The results of the long-term outcomes are provided in Additional file 1: S5. Patients randomized to haloperidol experienced fewer intrusive memories than the placebo group at hospital discharge (adjusted OR 0.40, 95% CI 0.40–0.40, *p* < 0.001). At 3 months after randomization, the haloperidol group was less likely to remember their ICU admission (adjusted OR 0.20, 95% CI 0.06–0.72, *p* = 0.014), and perceived their general health as better than the placebo group (adjusted difference 8.75, 95% CI 1.03–16.47, *p* = 0.032).

### Prespecified subgroup analyses

The prespecified subgroup analyses for motor subtype, presence of hallucinations or delusions, delirium severity, and delirium phenotype showed no statistically significant differences between groups (Additional file 1: Table E5).

### Post hoc outcomes

Patients who received haloperidol were less likely to fall or step out of bed than the placebo group (9% vs. 27%, aOR 0.32 [95% CI 0.11–0.84], *p* = 0.03), Table 3 and Additional file 1: Table E6. No other statistically significant differences were observed.

**Table 3:** Comparison of post hoc exploratory secondary outcomes between the haloperidol and placebo group

Outcome	Haloperidol (*n* = 65)	Placebo (*n* = 67)	Adjusted relative risk (95% CI)^a^	*p* value
No. of days with study drug, median (IQR)	4 (3–8)	6 (3–9)	0.96 (0.79–1.16)	0.66
No. of delirium days, median (IQR)	3 (2–6.5)	3 (2–5)	1.05 (0.81–1.37)	0.722
No. of coma days, median (IQR)	0 (0–2)	0 (0–1)	1.5 (0.85–2.66)	0.164
Agitation (RASS > 1), *n* (%)	25 (39)	30 (45)	OR: 0.84 (0.4–1.75)	0.638
No. of days, median (IQR)	0 (0–1)	0 (0–1)	0.77 (0.43–1.39)	0.388
Hallucinations/delusions, n (%)	55 (85)	51 (76)	OR: 1.75 (0.72–4.4)	0.220
No. of days, median (IQR)	2 (1–3)	3 (1–4.5)	0.74 (0.53–1.03)	0.075
Removal of urinary catheter, *n* (%)	5 (8)	9 (13)	OR: 0.48 (0.14–1.52)	0.226
Physical restraint, *n* (%)	48 (74)	48 (72)	OR: 1.19 (0.54–2.64)	0.660
(Almost) fell or stepped out of bed, *n* (%)	6 (9)	18 (27)	OR: 0.32 (0.11–0.84)	0.026

*CI* confidence interval, *IQR* interquartile range, *NA* not applicable, *OR* odds ratio, *RR* relative risk

^a^RR unless otherwise noted. The placebo group is used as reference

## Discussion

This multi-center randomized double-blind, placebo-controlled trial aiming to assess the effect of haloperidol on DCFDs within 14 days after randomization was preliminarily halted, per DSMB advice upon planned interim analysis because of futility of reaching the predefined difference of one day in DCFDs.

Our primary findings are in line with the MIND-USA and AID-ICU trial [7, 8]. However, the EuRIDICE trial differs from these prior trials in that it specifically assessed delirium outcomes related to agitation, which have not been previously reported in a therapeutic setting. Fewer haloperidol-treated patients required the use of a benzodiazepine, suggesting a benzodiazepine-sparing effect with haloperidol. Albeit non-significantly, other predefined secondary outcomes showed the same direction: study drug, rescue haloperidol, atypical antipsychotics and alpha-2 agonists use was lower in haloperidol-treated patients. The use of haloperidol was also significantly associated with the post hoc outcome of reduced patient harm associated with agitation (significantly fewer falls out of bed). The Hope-ICU trial, a trial assessing prophylactic effect of haloperidol, found that haloperidol reduced agitation [18], which is in line with our findings. An explanation could be that the EuRIDICE trial had a higher rate of agitated patients as compared to other trials (mixed delirium 73% in EuRIDICE versus 45% in AID-ICU [defined as RASS > 0] and 37% in MIND-USA with only 11% with agitated delirium upon inclusion) and included patients with lower disease severity (SOFA 5/6 vs. 11 in MIND-USA and mortality 16–21% as opposed to around 40% in the other two trials) [7, 8, 23].

Our findings, combined with those of Hope-ICU, encourage further research, focusing on delirium-associated symptoms, specifically those related to agitation, and the possible rescue medication-sparing effect of haloperidol rather than on delirium as a the main outcome of interest. More importantly, we feel that our results indicate that abandoning haloperidol from clinical practice to manage delirium symptoms at the ICU may not currently be warranted before further studies have definitely excluded such effects. Our findings and those from the Hope-ICU trial appear in line with current clinical experience, indicating that haloperidol might help reduce agitation in ICU patients with delirium.

Interestingly, no differences were observed regarding psychotic symptoms. It is unclear whether this points to haloperidol’s intrinsic lack of effect on psychotic features in ICU delirium, or the fact that more rescue atypical antipsychotics were administered in the control group. Another explanation may be the trial’s lack of statistical power.

Safety issues such as extrapyramidal symptoms, prolonged QTc interval and torsade de pointes occurred infrequently and did not differ between the two groups, consistent with the previous trials [7, 8]. We did, however, notice a significant decrease in blood pressure after haloperidol administration, but this safety measure did not appear to be clinically significant given the small drop in blood pressure.

A strength of this trial is that delirium assessments were performed three times daily. This may better facilitate the assessment of any response of fluctuating delirium symptoms to haloperidol compared with other studies [7, 24]. Other specific differences of EuRIDICE as opposed to MIND-USA and AID-ICU were: the possibility to use rescue atypical antipsychotics next to the study drug which was prohibited in both MIND-USA and AID-ICU; the exclusion of patients with possible alcohol related delirium, which was not specified in MIND-USA; the halting of study drugs when the patients were comatose (similar to MIND-USA), which was not advised in AID-ICU; and the assessment of many more relevant secondary outcomes related to delirium which is in line with a recent ICU delirium research core outcomes set [25]. Further, to our knowledge this is the first intervention study of haloperidol, administered to treat delirium while in the ICU, to report on a variety of patient-oriented long-term secondary outcomes. However, these findings are very preliminary as they concerned secondary outcomes from a preliminary halted, and consequently, underpowered clinical trial. However, our results may pave the way for further prospective research to determine possible efficacy of haloperidol to decrease the burden of recall of troublesome ICU experiences and memories and quality of life.

Our study has several limitations. First, this trial was prematurely terminated as advised by the DSMB partly because of randomization challenges due to the informed consent requirement (as compared to deferred consent in the AID-ICU trial), and therefore, in general all findings related to secondary outcomes should be viewed as hypothesis generating. Second, approximately 75% of all screened patients were deemed ineligible according to our exclusion criteria, which may limit external validity. Third, we did not assess actual adherence to the ABCDEF bundle during the intervention periods but only assessed estimates on adherence by the local PI’s [26]. Delirium assessments were performed by trained ICU nurses rather than dedicated study personnel. This may have influenced delirium diagnosis and management [27]. However, this trial was conducted in ICUs of which most participated in a large implementation study of delirium management [16], and in ICUs involved in a large delirium prevention study [28]. Therefore, these ICUs had sufficiently implemented routine delirium-oriented practices and are representative of real world clinical practice, supporting external validity of our study.

## Conclusion

This trial, that was stopped early, did not show evidence that haloperidol reduces delirium and coma in critically ill patients with delirium. The beneficial effects on some agitation-related outcomes and lower sedative requirements reported in a clinical trial are novel, clinically relevant and in line with clinical use. Together with some other signals of possible benefit in not previously reported (secondary) outcomes, these findings argue for additional effectiveness research of haloperidol for ICU delirium.

### Supplementary Information

**Additional file 1**. Online Data Supplement.

## Data Availability

All de-identified individual participant data that underlie the results reported in this article, and the data dictionary will be shared to investigators with the purpose of individual participant data meta-analysis after approval from the EuRIDICE steering committee and a signed data access agreement. All requests should be sent to m.vanderjagt@erasmusmc.nl. The study protocol is publicly available online.
