# Supplementary material for: Efficacy of haloperidol to decrease the burden of delirium in adult critically ill patients: the EuRIDICE randomized clinical trial
Source: Crit Care. 2023 Oct 30;27:413. doi: 10.1186/s13054-023-04692-3 (PMC10617114; doi:10.1186/s13054-023-04692-3)
Supplement: Supplementary file 1 — Additional file 1. Online Data Supplement. [file 13054_2023_4692_MOESM1_ESM.docx]

Online Data Supplement

**Efficacy of halopeRIdol to decrease the burden of Delirium In adult Critically ill patiEnts: The EuRIDICE Randomized Clinical Trial**
*Critical Care*

Lisa Smit, MD, Arjen J.C. Slooter, MD, PhD, John W. Devlin, PharmD, Zoran Trogrlic, RN, PhD, Nicole G.M. Hunfeld, PhD, Robert Jan Osse, MD, PhD, Huibert H. Ponssen, MD, Arjen J.B.W. Brouwers, MD, Jeannette F. Schoonderbeek, MD, PhD, Koen S. Simons, MD, PhD, Mark van den Boogaard, RN, PhD, Judith A. Lens, MD, Dirk P. Boer, MD, Diederik Gommers, MD, PhD, Wim J.R. Rietdijk, PhD, Mathieu van der Jagt, MD, PhD*, on behalf of the EuRIDICE study group.

*Corresponding author:

Department of Intensive Care, room Ne-415

Erasmus MC

PO BOX 2040

3000 CA Rotterdam, The Netherlands

E-mail: [m.vanderjagt@erasmusmc.nl](mailto:m.vanderjagt@erasmusmc.nl)

**Table of contents for the EuRIDICE Randomized Clinical Trial**

[Online Supplement 1. Data collection and definitions. 4](#_Toc147520755)

[Online Supplement 2. Quality ascertainment of delirium assessments and study procedures 7](#_Toc147520756)

[Online Supplement 3. Long-term outcomes. 17](#_Toc147520757)

[Online supplement 3 - Fig. 1 Flowchart of patients. 17](#_Toc147520758)

[Online supplement 3 - Table 1. Detailed follow-up rates of completed neuropsychological tests and questionnaires in eligible patients for each follow-up moment 17](#_Toc147520759)

[Table E1. Additional baseline characteristics of included patients. 19](#_Toc147520760)

[Table E2. Information related to delirium screening instrument. 20](#_Toc147520761)

[Figure E1. Adjusted number of delirium- and coma-free days during the 14-day intervention period. 21](#_Toc147520762)

[Figure E2. Distribution of the primary outcome delirium- and coma-free days in the haloperidol and placebo group. 22](#_Toc147520763)

[Online Supplement 4. Primary outcome; post-hoc analyses. 23](#_Toc147520764)

[Table E3. Additional comparison of predefined secondary outcomes between the haloperidol and placebo group. 24](#_Toc147520765)

[Table E4. Post-hoc analysis on the effect of haloperidol versus placebo on benzodiazepines among delirious ICU patients. 26](#_Toc147520766)

[Online Supplement 5 – Haloperidol and long-term outcomes 27](#_Toc147520767)

[Online Supplement 5 - Table 1. Haloperidol and wellbeing, memories and experiences in patients and family members after delirium in the ICU 27](#_Toc147520768)

[Online Supplement 5 - Table 2. Haloperidol and post-traumatic stress disorder symptoms 32](#_Toc147520769)

[Online Supplement 5 - Table 3. Haloperidol and anxiety and depression 33](#_Toc147520770)

[Online Supplement 5- Fig 1. The distribution of PTSD and anxiety and depression symptoms. 34](#_Toc147520771)

[Online Supplement 5 - Table 4. Haloperidol and long-term cognitive outcomes after delirium in critically ill patients, at 3 and 12 months after randomization in the ICU. 35](#_Toc147520772)

[Online Supplement 5 - Fig. 2 Differences in cognitive domains between the haloperidol and placebo group at 3 and 12 months after randomization 37](#_Toc147520773)

[Online Supplement 5 – Table 5. Difference in improvement of cognitive functioning between the haloperidol and placebo group from 3 to 12 months. 39](#_Toc147520774)

[Online Supplement 5 – Table 6. Haloperidol and long-term quality of life assessed with Short Form-36 after delirium in critically ill patients, at 3 and 12 months after randomization in the ICU. 40](#_Toc147520775)

[Online Supplement 5 - Fig. 3 SF-36 scores for haloperidol and placebo group at 3 (a) and 12 months (b) 41](#_Toc147520776)

[Online Supplement 5 - Table 7. Haloperidol and 1-year mortality after delirium. 42](#_Toc147520777)

[Table E5. Prespecified subgroup analyses: haloperidol versus placebo. 44](#_Toc147520778)

[Table E6. Additional comparison of post-hoc exploratory secondary outcomes between the haloperidol and placebo group. 45](#_Toc147520779)

[References 46](#_Toc147520780)

# Online Supplement 1. Data collection and definitions.

Data were collected in an online case record form, guided by the electronic data capture system of ALEA. We collected **baseline demographics at randomisation**, such as age, gender, ICU admission diagnosis, location before hospital (e.g. home with spouse, alone, rehabilitation facility) and before ICU admission (e.g. emergency department, hospital ward), presence of delirium at ICU admission, number of days of ICU admission before randomisation, QTc interval with 12-lead electrocardiography, Acute Physiology And Chronic Health Evaluation (APACHE) IV score, BMI, and clinical delirium phenotype, as previously defined.^1^

Each shift, trained ICU nurses evaluated each patient three times daily (once per 8-hour shift) for level of sedation with the Richmond Agitation Sedation Scale (RASS)^2^ and for delirium with either the Intensive Care Delirium Screening Checklist (ICDSC)^3^ or the Confusion Assessment Method for the ICU (CAM-ICU)^4^ if coma (RASS score -4 or -5) was not present. Each participating ICU used one of these delirium assessment methods consistently and non-interchangeably, as previously implemented.^5^ Three ICUs used the ICDSC and five used the CAM-ICU.

**During the intervention period, we recorded the following data** **on a daily basis (0:00-23:59)**: CAM-ICU or ICDSC and RASS scores of each 8-hour shift, sleep quality (assessed with a 7-point visual analogue scale by attending ICU nurse and sleep quality according to the patient with the Richards-Campbell Sleep Questionnaire^6^), maximum ICU Mobility Scale score^7^, modified Sequential Organ Failure Assessment (SOFA) score^8^ (without the central nervous system component) and intubation status (mechanical ventilation: ventilation with a tracheal tube for ≥3 hours; non-invasive mechanical ventilation: use of non-invasive methods, such as face masks, for ≥1 hours ).

**During the study period, patients were assessed daily** for **adverse drug associated events** (muscle-rigidity and associated movement disorders with the modified Simpson Angus Scale,^9^ akathisia with the Barnes Akathisia Rating Scale,^10^ and QTc prolongation using 12-lead electrocardiography once daily before study drug administration [>60msec above baseline or QTc ≥ 500ms]). Further, **adverse events related to delirium** (self-extubation, removal of invasive devices or catheters, physical restraints, [imminent or factual] falls out of bed), other **potential side effects of haloperidol** (epilepsy, tachycardia, unexplained hypotension, hepatic dysfunction, leucopenia and bronchospasms) and **serious adverse events were assessed** (SAEs: defined here as death, ventricular arrhythmia including torsade de pointes, or neuroleptic malignant syndrome).

We **further collected daily data regarding concomitant medication use**, next to the study drug and used for the treatment of hallucinations or agitation (“escape medication”). These included analgesics (including morphine, remifentanil, fentanyl, sufentanil, piritramide), sedative drugs (benzodiazepines [midazolam, tempazepam, oxazepam, lorazepam, bromazepam, zopiclone; administered both as continuous infusion or as intermittent bolus], propofol, clonidine, dexmedetomidine), open-label haloperidol and other antipsychotics (olanzapine, quetiapine, mirtazapine, clozapine, risperidone). The concomitant medication used was a predefined secondary outcome and was deemed important since differences in these drugs between intervention and placebo groups would indicate an effect of haloperidol that could contribute to further insights and hypotheses on specific effects of haloperidol, independent from any impact of the primary outcome. Opioids were reported as fentanyl equivalents (fentanyl = remifentanil^11^ = 20*morphine^11^ = 5* sufentanil^12^ = 30*piritramide^13^), and benzodiazepines as lorazepam equivalents (lorazepam 1 mg = midazolam 2.5 mg^11^ = zopiclone 3.75 mg = diazepam 5 mg^11^ = bromazepam 5mg^14^ = temazepam 10 mg^15^ = oxazepam 15mg^15^ = clonazepam 0.25mg). We did not differentiate between intravenous and intermittent benzodiazepine administration.

Regarding motoric delirium subtype, patients were classified as having **hyperactive delirium** if they had a RASS score +1 to +4 during all positive delirium assessments.^16^ **Hypoactive delirium** was classified when the RASS score was 0 to -3 at each positive delirium assessment, while **mixed delirium** was defined as either hyper- or hypoactive delirium during the 14-day intervention period. The **presence of psychotic symptoms** (hallucinations or delusions) in the centres using the ICDSC as delirium tool was based on the pertinent ICDSC criterion evaluating psychotic symptoms. In the centres using the CAM-ICU, this was based on whether or not ICU nurses had observed hallucinations or delusions, which they were required to report in an observation notebook for each shift. Delirium severity was only assessed for centres using the ICDSC as screening tool. Assuming that higher ICDSC scores indicate delirium severity ^17^, low delirium severity was defined as a mean ICDSC score of 4 to 5, medium delirium severity as a mean ICDSC score 5 to 7, and high delirium severity as a mean ICDSC score ≥7. **Delirium phenotypes** at randomisation (sedation-related, hypoxic, metabolic or septic delirium) were classified using previously published definitions.^1^ We defined sedation-related delirium as the administration of a sedative at the day of randomisation (either benzodiazepine, propofol, clonidine, opioids or dexmedetomidine).

Due to the premature termination, a cost-effectiveness analysis was not deemed feasible.

**Long-term outcomes**

Long-term outcomes (i.e. cognitive and functional outcomes, anxiety and depression, posttraumatic stress syndrome, memories and experiences, caregiver strain, 1-year mortality) were collected as followed. During the one-year follow-up, outcomes were obtained via postal questionnaires which were send out to patients and family members or during visits in an outpatient clinic, at the hospital or at the patients’ residence. If patients did not respond to the postal questionnaires, they were called to increase the response rate. Patients who did not respond to consecutive questionnaires were considered to be lost to follow-up. All assessments were blinded.

*Patient and family member wellbeing, memories and experiences associated with delirium and the ICU*

Patient and family member wellbeing, memories and experiences associated with delirium and the ICU were assessed with the ICU Memory Tool (ICU-MT) ^18^ and Delirium Experience Questionnaire (DEQ) ^19^. With the ICU-MT we collected whether patients remembered their hospital admission (clearly/hazily/not at all) and ICU stay (yes/no), the number of factual memories (i.e. alarms or presence of an endotracheal tube, score range 0 to 11), memories of feelings (i.e. feeling down or pain, score range 0 to 6) and delusional memories (i.e. hallucinations, score range 0 to 4) during ICU stay, and whether patients experienced panic or intrusive memories (yes/no). The DEQ consists of six questions for the patients, and one for caregivers or family members. We reported whether patients remembered their delirium, and in which extent this was burdensome (on a 5-point scale from 0 to 4, with 4 being the worst burden), and in which extent delirium was burdensome for family members on a similar scale. The ICU-MT and DEQ had not previously been translated to Dutch language. We used forward and backward translation to maintain equivalence of the questionnaires in Dutch.

*PTSD symptoms in patients and family members*

PTSD symptoms in patients and family members at 3 months after randomization were assessed with the Impact of Event Scale-Revised (IES-R) ^20^. The Dutch translation of the IES-R contained 22 questions related to PTSD symptoms caused by ICU stay, rated from 0 (not at all) to 4 (extremely). We reported the total score for each treatment group (ranging from 0 to 88, with 88 indicating the most severe PTSD) and the mean IES-R score, calculated by taking the mean of all questions. PTSD was defined as a mean IES-R score >= 1.6. We also calculated mean scores for the intrusion (8 questions), avoidance (8 questions) and hyperarousal subscales (6 questions).

*Burden experienced by the family*

Burden experienced by the family at 3 months after randomization was assessed with the Caregiver Strain Index (CSI) ^21^. Family members expressed their burden on the validated Dutch CSI, for which we reported the number of symptoms related to strain, with a score of ≥7 indicating that the family member experienced stress (maximum score 13).

*Cognition*

Cognition was assessed at 3 and 12 months after randomization by trained psychology professionals or study personnel who were blinded for the study drug with a detailed cognitive assessment battery of validated and repeatable measures of general cognition, memory, language, processing speed, attention and executive functioning (Montreal Cognitive Assessment [MoCA] ^22^, Rey Auditory Verbal Learning Test [RAVLT] ^23^, Semantic fluency ^24^, Digit Span [WAIS-IV] ^25^, Trail making tests [TMT] A and B ^26^, Boston naming Test [short version] ^27^). We accepted a time span of two weeks before and after the target date. All neuropsychological tests were validated Dutch versions and two versions were used to enable repeat testing of a patient. We described differences between the haloperidol and placebo group for six cognitive domains: 1) general cognition using the total MoCA score (maximum score 30 [patients with education <12 years obtained an extra score of 1], with higher scores indicating a better cognition and a score <26 considered as generally impaired cognition); 2) memory using the scores of the TAVLT related to relative memory capacity (total score of 5 trials, maximum score 75), delayed free-recall (maximum score 15) and recognition memory (maximum score 30, higher scores reflect better memory); 3) language as verbal fluency with the semantic fluency test (with higher scores indicating better fluency) and as confrontational naming abilities using the Boston Naming Test (short version, maximum score 30, with higher scores indicating better naming); 4) processing speed using the total points obtained at the WAIS-IV (maximum score 30, higher scores indicating better processing speed); 5) attention with the TMT Part A (maximum score 300 seconds, lower scores reflect better attention); and 6) executive functioning with the TMT Part B (maximum score 300 seconds, with lower scores reflecting better executive functioning).

*Anxiety and depression*

Anxiety and depression were assessed with the Hospital Anxiety and Depression Scale (HADS) at 3 and 12 months after randomization ^28^. The presence of anxiety or depression was defined as a total score of ≥8 on the respective subscales of the validated Dutch HADS (with a maximum score of 21 for each subscale).

*Quality of life*

Quality of life at 3 and 12 months after randomization was assessed with the Medical Outcome Study 36-Item Short Form Health Survey (SF-36) ^29^.Quality of life was assessed using nine subdomains of the validated Dutch version of the SF-36, related to physical functioning, role limitations due to physical health or emotional problems, energy/fatigue, emotional well-being, social functioning, pain, general health and health change, with a higher score on each subscale indicating a more favorable quality of life (range 0 to 100). A difference of five points between the haloperidol and placebo group is considered to be clinically and socially relevant ^29^.

*12-months mortality*

Mortality was assessed as a binary endpoint at 12 months after randomization, for which the hospital information system was used or information was obtained through family members or general practitioners.

# Online Supplement 2. Quality ascertainment of delirium assessments and study procedures

Participating ICUs either previously participated in a large implementation study on ICU delirium guidelines^5^ or were involved and experienced in previous research regarding ICU delirium.^30^ Site principal investigators and research nurses coordinated local study procedures after being trained and instructed in several meetings with the coordinating study team (LS, MvdJ, ZT). Standard clinical practice for the administration of haloperidol, analgesics and sedation was followed according to clinical protocols, based on the clinical practice guidelines from the Society of Critical Care Medicine.^31^ Spot-checks were performed and documented to confirm delirium assessment accuracy and quality, and to assess inter-observer agreement between ICU nurses and the local delirium researcher. Local physicians and ICU nurses, not primarily involved in the research team, were informed and locally trained in the study procedures, and a trial telephone was available 24/7 for any questions regarding the study or randomisation procedure.

**Spot-checks to confirm delirium assessment accuracy and quality: inter-observer agreement between ICU nurses and the local delirium researcher**

To assess the quality of delirium assessments, we requested each local research team to perform at least five spot-checks. With these spot-checks, we evaluated the quality of the CAM-ICU or ICDSC assessments by ICU nurses. After a patient was assessed for delirium by an ICU nurse, the same assessment was performed again by a member of the local research team or expert team (“delirium expert”). The delirium expert was unaware of the delirium assessment score obtained by the ICU nurse. Both delirium assessment scores (separate items and delirium diagnosis) were noted on a form. The scores from the ICU nurse and delirium expert were compared and potential differences and difficulties during the delirium assessment were discussed.

To assess inter-observer agreement, each item of the delirium assessment tools (CAM-ICU: 5 items; ICDSC: 8 items) and the overall score of the CAM-ICU (positive/negative) and ICDSC (delirium present if ≥4) were compared between the ICU nurse and the delirium expert. The spot-checks showed that there was good interobserver-agreement (≥90%) between the ICU nurse and delirium expert for both the CAM-ICU and ICDSC.

These spot-check were performed before start of the study in all centres, except in one centre which was involved in a large prophylactic haloperidol trial and consequently had ample experience with delirium assessment and management.^30^ Further, spot-checks were performed once more during the study in three centres (October 2019).

| **Delirium assessment tool** | **Agreement on separate items** | **Overall agreement on delirium diagnosis** | **Difficulties with delirium assessment** |
| --- | --- | --- | --- |
| CAM-ICU, n = 30 | 135/150 (90%) | 28/30 (93.3%) | - In patients with a language barrier, neurologic diagnosis (i.e. aphasia) or a sedation level of RASS -3 - Definition of fluctuation |
| ICDSC, n = 53 | 413/424 (97.5%) | 51/53 (96.2%) | - In sedated, non-responsive or hypoactive patients or patients with critical illness polyneuropathy or a language barrier - Assess sleep-wake cycle, orientation and presence of hallucinations |

**Assessment of adherence to pain, agitation and delirium guidelines**

To re-assess adherence to pain, agitation and delirium guidelines in the different participating ICUs, after the previous implementation study (see previous section) at the beginning of the trial, we requested local investigators to fill in a questionnaire. The questionnaire consisted of matters related to strategy (education, delirium assessment quality, study teams, pocket cards, reminders in electronic patient files and during handovers, etc.) and Pain, Agitation and Delirium (PAD) protocol (delirium prevention and treatment, evaluation of sedation, family involvement). These questions were answered at the start of the implementation phase of the trial logistics and right before start of inclusion. Each local research nurse and principal investigator answered these questions to their best knowledge of current practice at that time by giving a rating between 0 and 100% or ‘not applicable’.

We assessed the answers to the questionnaire after the implementation phase for the trial. Based on the results, general points for improvement consisted of obtaining a Good Clinical Practice (GCP) certificate for local researchers. Furthermore, it was necessary to determine who would be included in the local research and expert teams and to appoint local key-users. In addition, there were no pocket cards or educational sessions available for the EuRIDICE trial or delirium assessments. More attention was needed for delirium assessments and management, for example by performing spotchecks and by discussing delirium assessments during handovers.

Right before inclusion we performed another quality check using the answers the local investigators provided with the questionnaire. In general, we observed that the participating ICUs perceived improved adherence to the PAD guidelines, with more focus on delirium assessments and delirium management. Study teams and expert teams were set up with a specification of responsibilities during the trial. In addition, study teams and local physicians and nurses followed an online or classical course about the trial.

The answers for each center, after implementation phase (A) and right before inclusion (B), are found in the tables below. One center did not partake in the quality check; one center was enrolled during the randomization phase of the trial and therefore the answers for the implementation phase and right before inclusion collided.

**Questionnaire current care in the context of Implementation Readiness test**

| **Strategy** | **Subject** | **Question** | **NA** | **0%** | **20%** | **40%** | **60%** | **80%** | **100%** |
| --- | --- | --- | --- | --- | --- | --- | --- | --- | --- |
| Education | GCP certificate | The PI’s and RN have obtained their GCP certificate | A (4, 6) |  |  | A (1) | B (6) |  | A (3, 5, 7); B (1, 2, 3, 4, 5) |
|  | E-learning – Standard Operating Procedures | ≥80% of the nurses followed an e-learning | A (1, 2, 3, 4, 5, 6); B (6) | A (7) | B (3) | B (5) | B (4) | B (1) | A (2); B (2) |
|  | E-learning - Standard Operating Procedures | ≥80% of the physicians followed an e-learning | A (1, 2, 5, 6); B (4)3, 4, 6) | A (7); B (1) | B (3) |  | B (4, 5) |  | B (2) |
|  | E-learning - Standard Operating Procedures | All local principal investigators (PI’s) and research nurses (RN) followed an e-learning | A (1, 2, 3, 4, 5, 6) | A (7) | B (3) |  | B (4) |  | B (1, 2, 5, 6) |
|  | Clinical lesson informed consent | At least 1 member of the local study team (RN and/or PI and/or other members of the local team) took the lesson | A (1, 2, 3, 4, 5, 6) |  |  |  | B (1) |  | A (7); B (2, 3, 4, 5, 6) |
|  | Clinical lesson administering neurocognitive test before start inclusion | At least 1 member of the local study team (RN and/or PI and/or other members of the local team) took the lesson | A (1, 2, 3, 4, 5, 6, 7) | B (4) |  |  | B (1) |  | B (2, 3, 5, 6) |
|  | Clinical lesson administering neurocognitive test during follow-up period | At least 1 member of the local study team (RN and/or PI and/or other members of the local team) took the lesson | A (1, 2, 3, 4, 5, 6, 7); B (5) | B (4) |  |  |  |  | B (1, 2, 3, 6) |
|  | Clinical lesson (+ e-learning) filling in online CRF | At least the local RN, potentially also PI and/or other members of the local team, took the lesson | A (1, 2, 3, 4, 5, 6); B (1) | B (4, 6) | B (3, 5) | A (7) |  |  | B (2) |
|  | Clinical lesson or e-learning study procedures and protocol (via Erasmus MC pharmacy) | At least 1 member of the local study team (RN and/or PI and/or other members of the local team) took the lesson | A (1, 2, 3, 4, 5, 6); B (1) | B (4, 6) | B (3) |  |  |  | A (7); B (2, 5) |
| Quality screening | Quality screening check | Are the delirium scores checked by experts (“spotchecks”)? | A (3, 5, 7) | A (1, 2, 4) |  | A (6) |  | B (4) | B (1, 2, 3, 5, 6) |
| Expert team |  | Local expert team is multidisciplinary (*at least: intensivist, ICU nurse, physiotherapist*, and potentially: psychiatrist/neurologist/geriatrician) | A (3, 6) | A (1, 2) |  |  | B (6) | B (1) | A (4, 5, 7); B (2, 3, 5) |
|  |  | There has been at least 2 consultations between local expert team members (since the start of the study) and agreements have been made about implementation/the study | A (3, 4, 5, 6) | A (1, 2, 7) | B (3) | B (6) |  | B (4) | B (1, 2, 5) |
|  |  | It has been agreed (and documented) who is responsible and accountable for each specific part of the implementation process - see this IRT | A (3, 5, 6); B (6) | A (1, 4); B (1) | B (3) | A (7) |  | B (4, 5) | A (2); B (2) |
| Local study team |  | It is clear who have been appointed as expert team members; they serve as contact persons for delirium in general and the study in particular | A (3, 6) | A (1) | B (3) |  |  |  | A (2, 4, 5, 7); B (1, 2, 4, 5, 6) |
|  |  | A local reserve PI and RN have been designated | A (3, 7) | A (1); B (3) |  |  |  |  | A (2, 4, 5, 6); B (1, 2, 4, 5, 6) |
|  |  | Measures have been taken (e.g. through rosters) to designate a key user per shift during the inclusion period, especially during night shifts. This is to guarantee continuity for the study and the local procedures. | A (2, 3, 5, 6); B (2, 6) | A (1);  B (1, 3) | B (5) |  |  |  | A (4, 7); B (4) |
| Decision support | Laminated pocket cards screening with CAM-ICU or ICDSC | Pocket cards are present and available for the nurses and doctors | A (1, 2, 3, 4, 5, 6) | A (7); B (3, 4) |  |  |  | B (5) | B (1, 2, 6) |
|  |  | Pocket cards are used in practice | A (1, 2, 4, 5, 6); B (1) | A (7);B (4, 5) |  |  | B (3) | A (3); B (2) | B (6) |
| Reminders |  | There are reminders regarding screening and management of delirium (eg: 1) pop-ups PDMS for screening or 2) delirium included in daily visit form). | A (3) | A (5) |  | A (1) |  |  | A (6, 7); B (1, 2, 3, 4, 5, 6) |
| Focus groups/  barriers |  | Have bottlenecks been discussed in local multidisciplinary meetings in the center and is implementation aimed at addressing them? | A (1, 3, 4, 6) | A (5) | B (3) |  |  | B (2, 4) | A (2, 7); B (1, 5, 6) |

| **Protocol** | **Subject** | **Question** | **NA** | **0%** | **20%** | **40%** | **60%** | **80%** | **100%** | **Suggestion** |
| --- | --- | --- | --- | --- | --- | --- | --- | --- | --- | --- |
|  | PDMS | Is PDMS adapted and helpful in scoring the delirium assessment? |  | A (2, 5) |  |  |  | B (1) | A (1, 3, 4, 6, 7); B (2 ,3, 4, 5, 6) |  |
|  | Delirium treatment | With a new positive delirium screening, are the 4Hs + 4Ts used regularly? | A (7) | A (1, 4, 5); B (4, 5); B (6) |  |  | B (1) |  | A (2, 3); B (2, 3) |  |
|  |  | 1. Is medication ever adjusted as a result of the screening? 2. Is it clear what the pharmacological management of delirium is (protocol)? |  |  |  | A (1) | A (5) | A (6);B (5) | A (2, 3, 4, 7); B (1, 2, 3, 4, 6) |  |
|  |  | Is it checked before starting medication whether non-medicinal measures have already been optimized? |  |  |  | A (5); B (5) | A (4); B (6) | A (1, 6) | A (2, 3, 7); B (1, 2, 3, 4) |  |
|  | Prevention: Physiotherapy and early mobilization | Physio: have structural agreements been made and recorded with physio about early physiotherapy and mobilization? |  |  |  |  |  |  | A (1, 2, 3, 4, 5, 6, 7); B (2, 3, 4, 5, 6) |  |
|  |  | Is, in principle, attention paid daily during the visit to patient mobilization and has this been implemented in this visit? |  |  |  |  | B (1) | A (1, 5); B (5) | A (2, 3, 4, 6, 7); B (2, 3, 4, 6) |  |
|  |  | Is the department policy such that attempts are also made to mobilize ventilated patients next to bed (if possible)? |  |  | A (4) |  |  | A (1, 5); B (1, 5) | A (2, 3, 6, 7); B (2, 3, 4, 6) |  |
|  | Prevention: sleep hygiene | Is there a sleep promotion protocol? | A (7) | A (6); B (4) |  |  |  |  | A (1, 2, 3, 4, 5); B (1, 2, 3, 5, 6) |  |
|  |  | Is this protocol used and regularly followed in practice? | A (7) | A (6); B (4) |  |  |  | A (1, 5); B (5) | A (2, 3, 4); B (1, 2, 3, 6) |  |
|  |  | Does this protocol include at least: lights off or muted at night, striving for a good night's sleep (no regular visits if not necessary), and use of earplugs? | A (7) | A (6); B (4) |  |  |  |  | A (1, 2, 3, 4, 5); B (1, 2, 3, 5, 6) |  |
|  | Prevention: psychological hygiene (including reducing sensory deprivation) | Is there structural attention among all patients for the use of glasses/hearing aids if the patient normally uses them (during the day)? |  |  |  |  |  | A (1, 2) | A (3, 4, 5, 6, 7); B (1, 2, 3, 4, 5, 6) |  |
|  | Evaluation of pain-sedation-delirium | Daily screening delirium introduced and “running well”? |  |  | A (4) |  | A (1) | A (5); B (1) | A (2, 3, 6, 7); B (2, 3, 4, 5, 6) |  |
|  |  | Is attention to coordination of delirium, sedation and pain management implemented in any way in the visit (eg visit form)? |  |  |  | A (6) |  | A (1, 5); B (6) | A (2, 3, 4, 7); B (1, 2, 3, 5) |  |
|  |  | Is a visit (or MDO) checklist used? | A (7) | A (4, 6); B (4) |  | A (5); B (5) |  |  | A, 4 (1, 2, 3); B (1, 2, 3, 6) |  |
|  | Sedation | In principle, is midazolam (as much as possible/preferably) per continuous infusion avoided with sedation and alternative sedation used? (i.e. analgo-sedation with opiate and possibly clonidine/dexmedetomidine/propofol aimed at approachable comfortable patient) |  |  |  |  |  | A (2) | A (1, 3, 4, 5, 6, 7); B (1, 2, 3, 4, 5, 6) |  |
| Family | Folder | Is there a leaflet delirium for family? |  | B (4) |  |  |  |  | A (2, 3, 4, 5, 6, 7); B (1, 2, 3, 5, 6) |  |
|  |  | Is the family offered the opportunity to contribute to the identification and/or co-treatment of delirium (eg help with washing, etc.)? | B (2) | A (4); B (4) | A (5) | A (6); B (5) | B (6) | A (3) | A (1, 2, 7); B (1, 3) |  |
|  | Poster | The poster is hung up in the family room | A (1, 4, 7); B (1, 2, 5, 6) | A (5, 6); B (3, 4) |  |  |  |  |  |  |

# **Online Supplement 3. Long-term outcomes.**

## Online supplement 3 - Fig. 1 Flowchart of patients.

Sixteen patients (9 haloperidol, 7 placebo) who were not assessed at hospital discharge were tested at 3-month follow-up. Seven patients (4 haloperidol, 3 placebo) who were not assessed at 3-month follow-up were tested at 12-month follow-up.

In total, 68 patients participated in at least one follow-up moment (33 haloperidol, 35 placebo).

## Online supplement 3 - Table 1. Detailed follow-up rates of completed neuropsychological tests and questionnaires in eligible patients for each follow-up moment

| **Test** | **At hospital discharge** | | **At 3 months** | | **At 12 months** | |
| --- | --- | --- | --- | --- | --- | --- |
|  | **Haloperidol (n = 45)** | **Placebo (n = 46)** | **Haloperidol (n = 35)** | **Placebo (n = 36)** | **Haloperidol (n = 24)** | **Placebo (n = 25)** |
| Neurocognitive test, n (%) | - | - | 16 (46) | 17 (47) | 9 (38) | 11 (44) |
| ICU-MT, n (%) | 23 (45) | 26 (46) | 21 (60) | 22 (61) | - | - |
| DEQ, n (%) | 23 (51) | 26 (57) | 21 (60) | 21 (58) | - | - |
| CSI, n (%) | - | - | 21 (60) | 20 (56) | - | - |
| IES-R patient, n (%) | - | - | 19 (54) | 21 (58) | - | - |
| IES-R family, n (%) | - | - | 19 (54) | 16 (44) | - | - |
| HADS, n (%) | - | - | 16 (46) | 20 (56) | 17 (71) | 20 (80) |
| SF-36, n (%) | - | - | 18 (51) | 24 (67) | 17 (71) | 18 (72) |

CSI: Caregiver Strain Index; DEQ: Delirium Experience Questionnaire; HADS: Hospital Anxiety and Depression Scale; IES-R: Impact of Event Scale-Revised; ICU-MT: ICU Memory Tool; SF-36: Short Form-36.

Patients were deemed eligible if they still partook in the follow-up phase (i.e. were still alive, had not withdrawn consent and were not lost to follow-up). Hence, the percentages reflect the response rate to questionnaires. In total, 68 patients were evaluated during at least one follow-up moment.

# **Table E1. Additional baseline characteristics of included patients.**

| **Characteristic** | **Haloperidol**  **(n = 65)** | **Placebo**  **(n = 67)** |
| --- | --- | --- |
| Location before hospitalization | | |
| Home with spouse or with need for extra daily help from caretakers, n (%) | 39 (60) | 44 (66) |
| Home alone, n (%) | 15 (23) | 16 (24) |
| Rehabilitation facility, n (%) | 1 (2) | 0 (0) |
| Other or unknown, n (%) | 10 (15) | 7 (10) |
| Delirium phenotype present at randomization ^a^ | | |
| Sedation-related, n (%) | 58 (89) | 60 (90) |
| Sepsis, n (%) | 50 (77) | 48 (72) |
| Metabolic, n (%) | 21 (32) | 24 (36) |
| Hypoxia, n (%) | 10 (15) | 8 (12) |
| Delirium before ICU admission (prevalent delirium), n (%) | 3 (5) | 8 (12) |
| No. of days with delirium before ICU admission, median (IQR) | 1 (1 to NA) ^b^ | 2 (1 to 3.8) |
| Mean daily mSOFA score during the intervention period, median (IQR) | 3.9 (2.6 to 7.0) | 4.4 (3.4 to 6.9) |
| BMI, mean (SD) | 27.1 (5.4) | 26.6 (4.7) |

BMI = Body Mass Index; ICU = Intensive Care Unit; mSOFA = modified Sequential Organ Failure Assessment (without the central nervous system component); SOFA = Sequential Organ Failure Assessment

^a^ Patients could have multiple delirium phenotypes at randomization^1^, thus the sum of phenotypes may be greater than the total number of patients.

^b^ Due to the low number of events, an IQR could not be calculated. Of the 3 patients in the haloperidol group who experienced delirium before ICU admission, the number of days with delirium before admission were 1 (n=2) and 2 (n=1).

# **Table E2. Information related to delirium screening instrument.**

|  | **Total** | **Delirium screening instrument used** | |
| --- | --- | --- | --- |
|  |  | **ICDSC** | **CAM-ICU** |
| Screened patients, n (%) | 8075 | 4839 (60) | 3236 (40) |
| Eligible patients, n (%) | 1805 | 806 (45) | 999 (55) |
| Written informed consent obtained, n (%) | 290 | 207 (71) | 83 (29) |
| Randomized patients with delirium, n (%) | 132 | 105 (80) | 27 (20) |

# Figure E1. Adjusted number of delirium- and coma-free days during the 14-day intervention period.


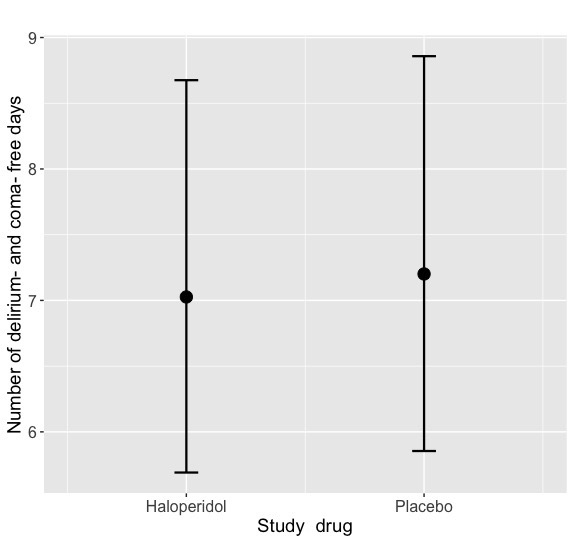


Based on a negative binomial mixed-effect model, adjusted for mSOFA at randomisation and hospital as a random effect, this graph displays the adjusted number of delirium- and coma-free days during the 14-day intervention period for the haloperidol and placebo group.

# Figure E2. Distribution of the primary outcome delirium- and coma-free days in the haloperidol and placebo group.


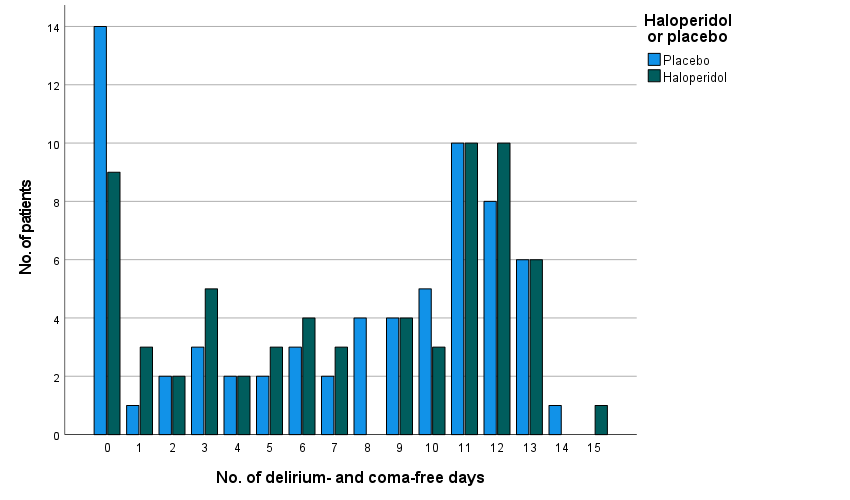


This graph displays the distribution of number of delirium- and coma-free days (DCFDs) during the 14-day intervention period for the haloperidol and placebo group. No data were missing.

# Online Supplement 4. Primary outcome; post-hoc analyses.

In order to increase comparability, we used similar definitions as the MIND-USA study (no. of DCFDs = 14 - delirium days - coma days - days deceased),^11^ thus not assuming that deceased patients had 0 DCFDs. This showed that the median number of DCFDs in the haloperidol group was 10 (IQR 3.5 to 12) and in the placebo group 9 (IQR 4 to 11), p = 0.58, with an adjusted RR of 1.00 (95%CI 0.79 to 1.27, p = 0.99).

Further, we assessed the impact on the number of DCFD of the assumption that patients who were discharged alive from the ICU were considered to be delirium- and coma-free after ICU discharge, regardless of their delirium status at discharge. Therefore, we left out this assumption. This yielded the following results: the median number of DCFDs in the haloperidol group was 2 (IQR 0 to 4**.**5) and in the placebo group 2 (0 to 5), with an adjusted RR of 1**.**15 (0**.**75 to 1**.**74, p = 0**.**53).

# Table E3. Additional comparison of predefined secondary outcomes between the haloperidol and placebo group.

| **Outcome** | **Haloperidol (n = 65)** | **Placebo (n = 67)** | **Adjusted difference (95%CI)** | **Adjusted relative risk (95%CI) ^a^** | **p value** |
| --- | --- | --- | --- | --- | --- |
| *Secondary outcomes: medication-related outcomes* | | | | | |
| Use of “escape” medication, no. of days, median (IQR) | 5 (3 to 11.5) | 6 (4 to 11) |  | 1.02 (0.81 to 1.28) | 0.884 |
| Olanzapine, mean 24h dose in mg, median (IQR) ^b^ | 0 | 5.8 (5 to 9.4) | -3.19 (-8.71 to 2.33) |  | 0.341 |
| Quetiapine, mean 24h dose in mg, median (IQR) ^b^ | 25  (12.5 to 38) | 28.6  (18.2 to 49.1) | -9.83 (-23.39 to 3.63) |  | 0.163 |
| Clonidine, mean 24h dose in mcg, median (IQR) ^b^ | 864.7  (573 to 1233.8) | 650.1  (381.4 to 1015.8) | 198.85 (-41.5 to 469.61) |  | 0.120 |
| Dexmedetomidine, mean 24h dose in mcg, median (IQR) ^b^ | 841.3  (224.5 to 7751.5) | 1707.8  (948 to 2853.5) | 637.19 (-5939.27 to 7534.67) |  | 0.854 |
| Benzodiazepine, mean 24h dose in mg, median (IQR) ^b, c^ | 2 (1.5 to 2.4) | 2 (1.4 to 3.2) | -19.1% (-48.5% to 28.8%) ^d^ |  | 0.368 |
| Propofol, mean 24h dose in mg, median (IQR) ^b^ | 1500  (739.5 to 3053.3) | 1165  (550 to 2362.4) | 283.34 (-304.84 to 950.41) |  | 0.377 |
| Opioid, mean 24h dose in mg, median (IQR) ^b, e^ | 50 (5 to 211.1) | 9.1 (2.8 to 101.1) | 127% (1.8% to 407.3%) ^d^ |  | 0.048 |
| *Secondary outcomes: safety outcomes* | | | | | |
| QTc prolongation, no. of episodes, median (IQR) ^b^ | 1 (1 to NA) | 1 (1 to 3.3) |  | 1.66 (0.44 to 6.24) | 0.455 |
| Muscle rigidity and associated movement disorders, no. of episodes, median (IQR) ^b^ | 1 (1 to 1) | 1 | - | - | - |
| Ventricular arrhythmia, no. of episodes, median (IQR) ^b^ | 1 (1 to 1) | 1 | - | - | - |

CI = confidence interval; DCFD = delirium- and coma-free day; IQR = interquartile range; OR = odds ratio; RR = relative risk.

^a^ RR unless mentioned otherwise. The placebo group was used as a reference. The RR may be interpreted as follows: the number of DCFDs in the haloperidol group is 0.98 times the number in the placebo group.

^b^ Reported only for patients in whom this medication was ever administered or in whom the event ever occurred during the study period.

^c^ Reported in lorazepam equivalents

^d^ After log transformation due to non-normality and/or heteroscedasticity, and needs to be interpreted as adjusted percent change for the haloperidol vs. placebo group. For example, compared to placebo, haloperidol decreases the median time to first resolution of delirium in days by 16.6 percent (adjusted percent change for haloperidol vs. placebo is -16.6%, not significant) if all other variables are kept constant.

^e^ Reported in fentanyl equivalents

# Table E4. Post-hoc analysis on the effect of haloperidol versus placebo on benzodiazepines among delirious ICU patients.

| **Outcome** | **Haloperidol**  **(n = 65)** | **Placebo**  **(n = 67)** | **Adjusted difference (95%CI)** | **Adjusted odds ratio (95%CI) ^a^** | **p value** |
| --- | --- | --- | --- | --- | --- |
| Continuous IV benzodiazepines, ever, n (%) | 7 (11) | 14 (21) |  | 0.49 (0.17 to 1.33) | 0.17 |
| Continuous IV benzodiazepines, median (IQR), days ^b^ | 1 (1 to 3) | 1 (1 to 2) |  | RR: 1.19 (0.51 to 2.79) | 0.69 |
| Continuous IV benzodiazepines, mean 24h dose in mg, median (IQR) ^b^ | 34 (2.5 to 76) | 29 (5 to 178.2) | -66.14 (-163.82 to 31.53) |  | 0.21 |
| Intermittent IV / p.o. benzodiazepines, ever, n (%) | 35 (54) | 44 (66) |  | 0.51 (0.24 to 1.07) | 0.08 |
| Intermittent IV / p.o. benzodiazepines, median (IQR), days ^b^ | 4 (1 to 6) | 3 (2 to 5) |  | RR: 1.16 (0.84 to 1.6) | 0.38 |
| Intermittent IV / p.o. benzodiazepines, mean 24h dose in mg, median (IQR) ^b^ | 2 (2 to 3.4) | 2 (1.7 to 3) | 0.24 (-0.31 to 0.79) |  | 0.41 |

CI = Confidence Interval; HR = hazard ratio; ICU = Intensive Care Unit; NA = not applicable (due to uneven low number of patients); RR = relative risk; SD = standard deviation

Continuous IV administered benzodiazepines included midazolam, and doses are shown in midazolam doses. Intermittently IV / p.o. administered benzodiazepines included lorazepam, zopiclone, diazepam, bromazepam, temazepam, oxazepam, and clonazepam, and doses are shown in lorazepam equivalents.

^a^ OR unless mentioned otherwise. Placebo group was used as a reference. A RR of 2.5, for instance, may be interpreted as that an event occurred at 2.5 times the rate in the haloperidol group than in the placebo group.

^b^ Calculated only for days on which mentioned benzodiazepines were administered during the 14-day intervention period (excluding days without administration).

# Online Supplement 5 – Haloperidol and long-term outcomes

## Online Supplement 5 - Table 1. Haloperidol and wellbeing, memories and experiences in patients and family members after delirium in the ICU

| **Wellbeing, memories and experience** | **Haloperidol** | **Placebo** | **Unadjusted OR (95%CI)** | **Unadjusted difference (95%CI) ^a^** | **p value** | **Adjusted OR (95% CI)** | **Adjusted difference (95% CI) ^b^** | **p value** |
| --- | --- | --- | --- | --- | --- | --- | --- | --- |
| *At hospital discharge* | | | | | | | | |
| *ICU-MT (haloperidol: n = 23; placebo: n = 26)* | | | | | | | | |
| Remembers hospital admission | | | | | | | | |
| Clearly, n (%) | 11 (48) | 14 (54) | 0.79 (0.25 to 2.42) |  | 0.674 | 0.79 (0.26 to 2.42) | - | 0.674 |
| Hazily, n (%) ^c^ | 4 (17) | 4 (15) | 1.16 (0.24 to 5.51) |  | 0.850 | - | - | - |
| Not at all, n (%) | 8 (35) | 8 (31) | 1.20 (0.36 to 4.03) |  | 0.765 | 1.20 (0.36 to 3.97) | - | 0.765 |
| Remembers ICU admission, n (%) ^d^ | 12 (55) | 16 (64) | 0.68 (0.21 to 2.18) |  | 0.511 | 0.68 (0.21 to 2.18) | - | 0.511 |
| No. of factual memories, median (IQR) | 3.0 (1.0 to 7.0) | 4.0 (1.0 to 8.0) |  | -0.94 (-2.82 to 0.93) | 0.316 |  | -1.17 (-2.84 to 0.60) | 0.180 |
| No. of memories of feelings, median (IQR) | 2.0 (0.0 to 3.0) | 2.5 (0.8 to 4.0) |  | -0.36 (-1.32 to 0.61) | 0.463 |  | -0.67 (-1.53 to 0.34) | 0.141 |
| No. of delusional memories, median (IQR) | 1.0 (0.0 to 3.0) | 2.0 (0.0 to 2.3) |  | 0.10 (-0.66 to 0.86) | 0.785 |  | 0.10 (-0.64 to 0.84) | 0.785 |
| Remembers delusional memories only, n (%) ^c^ | 1 (4) | 0 (0) | 3.53 (0.14 to 91.09) ^e^ |  | 0.446 | - | - | - |
| Remembers factual events only, n (%) ^c^ | 1 (4) | 2 (8) | 0.55 (0.02 to 6.08) |  | 0.630 | - | - | - |
| Remembers delusions, feelings and factual events, n (%) | 13 (57) | 14 (54) | 1.11 (0.36 to 3.49) |  | 0.851 | 0.66 (0.17 to 2.57) | - | 0.545 |
| Remembers nothing, n (%) ^c^ | 3 (13) | 3 (12) | 1.15 (0.19 to 6.83) |  | 0.873 | - | - | - |
| Experienced panic, n (%) ^d^ | 7 (32) | 12 (46) | 0.54 (0.16 to 1.75) |  | 0.314 | 0.54 (0.17 to 1.78) | - | 0.314 |
| Experienced intrusive memories, n (%) | 4 (17) | 8 (31) | 0.47 (0.11 to 1.78) |  | 0.282 | 0.40 (0.40 to 0.40) | - | <.001 |
| *DEQ (haloperidol: n = 23; placebo: n = 26)* | | | | | | | | |
| Remembers delirium, n (%) | 9 (39) | 14 (54) | 0.55 (0.17 to 1.70) |  | 0.305 | 0.55 (0.18 to 1.72) | - | 0.305 |
| Burden of remembering delirium, median (IQR) ^d^ | 2.5 (1.0 to 3.0) | 3.0 (1.0 to 4.0) |  | -0.32 (-1.68 to 1.03) | 0.626 |  | -0.32 (-1.59 to 0.95) | 0.626 |
| Burden of not remembering delirium, median (IQR) ^d^ | 0.0 (0.0 to 2.0) | 0.0 (0.0 to 2.0) |  | -0.06 (-1.00 to 0.87) | 0.890 |  | -0.186 (-0.94 to 0.82) | 0.672 |
| Family: burden of observing delirium, median (IQR) | 3.0 (2.0 to 3.0) | 2.5 (1.0 to 3.3) |  | 1.14 (-12.68 to 14.96) | 0.869 |  | 0.16 (-10.57 to 10.93) | 0.977 |
| *3 months after randomization* | | | | | | | | |
| *ICU-MT (haloperidol: n = 23; placebo: n = 22)* | | | | | | | | |
| Remembers hospital admission | | | | | | | | |
| Clearly, n (%) | 11 (48) | 7 (32) | 1.96 (0.59 to 6.86) |  | 0.276 | 1.96 (0.58 to 6.61) | - | 0.276 |
| Hazily, n (%) | 5 (22) | 5 (23) | 0.94 (0.22 to 3.96) |  | 0.937 | 0.94 (0.23 to 3.85) | - | 0.937 |
| Not at all, n (%) | 7 (30) | 10 (46) | 0.53 (0.15 to 1.76) |  | 0.301 | 0.53 (0.15 to 1.78) | - | 0.301 |
| Remembers ICU admission, n (%) | 6 (26) | 14 (64) | 0.20 (0.05 to 0.69) |  | 0.014 | 0.20 (0.06 to 0.72) | - | 0.014 |
| No. of factual memories, median (IQR) ^d^ | 3.0 (1.8 to 5.3) | 5.0 (2.0 to 6.5) |  | -0.98 (-2.77 to 0.81) | 0.277 |  | -0.98 (-2.71 to 0.76) | 0.277 |
| No. of memories of feelings, median (IQR) ^d^ | 1.0 (0.0 to 3.0) | 2.0 (1.5 to 4.0) |  | -0.70 (-1.78 to 0.37) | 0.194 |  | -0.70 (-1.75 to 0.34) | 0.194 |
| No. of delusional memories, median (IQR) ^d^ | 1.0 (0.0 to 2.0) | 1.0 (1.0 to 2.5) |  | -0.44 (-1.19 to 0.31) | 0.243 |  | -0.42 (-1.16 to 0.29) | 0.253 |
| Remembers delusional memories only, n (%) ^c^ | 1 (5) | 0 (0) | 3.00 (0.12 to 77.65) ^e^ |  | 0.508 |  | - | - |
| Remembers factual events only, n (%) ^c, d^ | 3 (14) | 0 (0) | 7.68 (0.37 to 157.92) ^e^ |  | 0.186 |  | - | - |
| Remembers delusions, feelings and factual events, n (%)  ^d^ | 12 (55) | 15 (71) | 0.48 (0.13 to 1.67) |  | 0.256 | 0.47 (0.12 to 1.85) | - | 0.282 |
| Remembers nothing, n (%) ^c, d^ | 3 (14) | 0 (0) | 7.68 (0.37 to 157.92) ^e^ |  | 0.186 |  | - | - |
| Experienced panic, n (%) | 5 (22) | 8 (36) | 0.49 (0.12 to 1.78) |  | 0.283 | 0.49 (0.13 to 1.82) | - | 0.283 |
| Experienced intrusive memories, n (%) ^c^ | 3 (13) | 4 (18) | 0.68 (0.12 to 3.46) |  | 0.636 | - | - | - |
| *DEQ (haloperidol: n = 23; placebo: n = 21)* | | | | | | | | |
| Remembers delirium, n (%) | 11 (48) | 15 (71) | 0.37 (0.10 to 1.25) |  | 0.116 | 0.37 (0.10 to 1.28) | - | 0.116 |
| Burden of remembering delirium, mean (SD) | 2.6 (1.4) | 1.8 (1.4) |  | 0.76 (-0.46 to 1.97) | 0.211 |  | 0.76 (-0.39 to 1.90) | 0.211 |
| Burden of not remembering delirium, median (IQR) | 0.0 (0.0 to 2.0) | 0.0 (0.0 to 1.5) |  | 0.22 (-1.12 to 1.56) | 0.732 |  | 0.28 (-0.77 to 1.26) | 0.583 |
| Family: burden of observing delirium, median (IQR) | 2.0 (0.0 to 3.0) | 2.0 (1.0 to 3.0) |  | -36.84% (-71.02% to 37.66%) ^f^ | 0.239 |  | -36.84% (--71.02% to 37.66%) ^f^ | 0.239 |
| *Family member / caregiver (haloperidol: n = 21; placebo: n = 20)* | | | | | | | | |
| CSI score, mean (SD) | 5.8 (3.8) | 6.5 (2.6) |  | -0.69 (-2.75 to 1.37) | 0.503 |  | -0.69 (-2.68 to 1.31) | 0.503 |
| Presence of significant stress (CSI score ≥7), n (%) | 11 (52) | 11 (55) | 0.90 (0.26 to 3.09) |  | 0.867 | 0.90 (0.26 to 3.07) | - | 0.867 |

CSI: Caregiver Strain Index (maximum score is 12, higher scores indicate more caregiver strain); DEQ: Delirium Experience Questionnaire (burden is expressed on a 5-point Likert scale, with high scores indicating a higher burden); ICU-MT: ICU Memory Tool; OR: Odds Ratio

The median time until the questionnaires were sent back was 41 days (IQR 21.5 to 59.5) after randomization for the ICU-MT and 40 days (IQR 19.5 to 59.5) for the DEQ at hospital discharge, and 96 days (IQR 83.5 to 114.5) for the ICU-MT and 96 days (IQR 82.8 to 114.8) for the DEQ at the 3-month follow-up.

^a^ Continuous variables are expressed as differences (β estimate) in scores between the haloperidol and placebo group (reference), and categorical variables as OR, with placebo being the reference group.

^b^ Adjusted with a random effect for hospital. There were no baseline differences between the haloperidol and placebo group.

^c^ Adjusted analyses were not possible due to the low number of events or number of patients with this specific outcome (10 events / patients required for one predictor).

^d^ Some values were missing: remembers ICU admission at discharge 2 (4%), experienced panic at discharge 1 (2%), burden of not remembering delirium at discharge 2 (4%), burden of remembering delirium at discharge 1 (2%), remembers only delusional memories at 3 months 2 (4%), remembers only factual memories at 3 months 2 (4%), remembers nothing at 3 months 2 (4%), remembers delusions, feelings and factual events 2 (4%), no. of factual memories at 3 months 2 (4%), no. of memories of feelings at 3 months 2 (4%), no. of delusional memories at 3 months 2 (4%).

^e^ Due to zero (non-)cases/events of the outcome in this comparison group, unadjusted odds ratio were calculated by applying a Haldane-Anscombe correction (adding 0.5 to all cells in the contingency table).

^f^ After log transformation due to non-normality and/or heteroscedasticity, and hence needs to be interpreted as adjusted percent change for the haloperidol group vs. placebo group. This was calculated by exponentiating the log-transformed coefficient, and subsequently subtracting one from that obtained number and multiplying this with 100.

## Online Supplement 5 - Table 2. Haloperidol and post-traumatic stress disorder symptoms

| **IES-R outcomes** | **Haloperidol** | **Placebo** | **Unadjusted difference (95%CI) ^a^** | **p value** | **Adjusted difference (95% CI) ^a, b^** | **p value** |
| --- | --- | --- | --- | --- | --- | --- |
| *Patient (haloperidol: n = 19; placebo: n = 21)* | | | | |  |  |
| IES-R total score, median (IQR) | 13.0 (3.0 to 30.0) | 12.0 (0.5 to 17.5) | 1.90 (-6.85 to 10.64) | 0.663 | 1.90 (--6.56 to 10.35) | 0.663 |
| IES-R mean score, median (IQR) | 0.6 (0.1 to 1.4) | 0.5 (0.0 to 0.8) | 0.09 (-0.31 to 0.48) | 0.663 | 0.09 (-0.30 to 0.47) | 0.663 |
| Presence of PTSD, n (%)^c^ | 0 (0) | 3 (14) | OR: 0.14 (0.01 to 2.81) ^d^ | 0.196 | - | - |
| Intrusion score, median (IQR) | 0.5 (0.1 to 1.0) | 0.4 (0.1 to 0.9) | -0.01 (-0.51 to 0.49) | 0.957 | -0.01 (-0. 50 to 0.47) | 0.957 |
| Avoidance score, median (IQR) | 0.4 (0.1 to 0.9) | 0.5 (0.0 to 1.0) | 0.00 (-0.42 to 0.42) | 0.996 | 0.00 (-0.40 to 0.40) | 0.996 |
| Hyperarousal score, median (IQR) | 0.7 (0.0 to 1.2) | 0.2 (0.0 to 0.8) | 0.33 (-0.10 to 0.77) | 0.127 | 0.33 (-0.08 to 0.75) | 0.127 |
| *Family member / caregiver (haloperidol: n = 19; placebo: n = 16)* | | | | |  |  |
| IES-R total score, median (IQR) | 14.0 (2.0 to 39.0) | 17.0 (4.8 to 26.8) | -0.61 (-14.25 to 13.04) | 0.928 | -0.61 (-13.73 to 12.51) | 0.928 |
| IES-R mean score, median (IQR) | 0.6 (0.1 to 1.8) | 0.8 (0.2 to 1.2) | -0.03 (-0.65 to 0.59) | 0.928 | -0.03 (-0.62 to 0.57) | 0.928 |
| Presence of PTSD, n (%) | 5 (26) | 3 (19) | OR: 1.55 (0.31 to 8.79) | 0.597 | - | - |
| Intrusion score, median (IQR) | 0.9 (0.3 to 2.5) | 0.9 (0.3 to 2.1) | 0.02 (-0.73 to 0.77) | 0.964 | 0.02 (-0.70 to 0.74) | 0.964 |
| Avoidance score, median (IQR) | 0.3 (0.0 to 1.4) | 0.4 (0.0 to 0.8) | 0.06 (-0.46 to 0.58) | 0.817 | 0.06 (0.44 to 0.56) | 0.817 |
| Hyperarousal score, median (IQR) | 0.7 (0.0 to 1.7) | 0.9 (0.0 to 1.8) | -0.20 (-0.93 to 0.52) | 0.571 | -0.20 (-0.90 to 0.49) | 0.571 |

IES-R: Impact of event scale revised (high score associated with PTSD-related symptoms); OR: odds ratio; PTSD: Post-traumatic stress disorder.

The median time until the questionnaires were sent back was 96 days (IQR 85.5 to 107.5) after randomization for the patients and 101 (IQR 88 to 114) for the family members.

^a^ Expressed as differences (β estimate) in scores between the haloperidol and placebo group (reference), unless mentioned otherwise.

^b^ Adjusted for a random effect for hospital. There were no baseline differences between the haloperidol and placebo group.

^c^ Adjusted analyses were not possible due to risk of overfitting (low number of patients/events relative to the number of covariates).

^d^ Due to zero (non-)cases/events of the outcome in one of the treatment groups, unadjusted odds ratio were calculated by applying a Haldane-Anscombe correction (adding 0.5 to all cells in the contingency table).

## Online Supplement 5 - Table 3. Haloperidol and anxiety and depression

| **HADS subdomain** | **Haloperidol** | **Placebo** | **Unadjusted difference / OR (95%CI) ^a^** | **p value** | **Adjusted difference / OR (95%CI) ^b^** | **p value** |
| --- | --- | --- | --- | --- | --- | --- |
| *At 3 months (haloperidol: n = 16; placebo: n = 20)* | | | | |  |  |
| Anxiety score, median (IQR) | 3.5 (1.3 to 5.0) | 3.0 (2.0 to 9.0) | -0.80 (-3.25 to 1.65) | 0.511 | -0.80 (-3.16 to 1.56) | 0.515 |
| Anxiety score ≥ 8, n (%) ^c^ | 2 (13) | 6 (30) | OR: 0.33 (0.04 to 1.74) | 0.222 | - | - |
| Depression score, median (IQR) | 3.0 (1.0 to 6.0) | 5.0 (3.0 to 7.8) | -1.35 (-3.67 to 0.97) | 0.245 | -1.28 (-3.58 to 0.88) | 0.262 |
| Depression score ≥ 8, n (%) ^c^ | 3 (19) | 5 (25) | OR: 0.69 (0.12 to 3.39) | 0.655 | - | - |
| *At 12 months (haloperidol: n = 17; placebo: n = 20)* | | | | |  |  |
| Anxiety score, median (IQR) | 6.0 (2.5 to 10.0) | 3.0 (1.0 to 6.0) | 1.76 (-1.12 to 4.64) | 0.223 | 1.81 (-1.02 to 4.53) | 0.207 |
| Anxiety score ≥ 8, n (%) | 6 (35) | 4 (20) | OR: 2.18 (0.51 to 10.33) | 0.302 | OR: 2.18 (0.50 to 9.58) | 0.302 |
| Depression score, median (IQR) | 4.0 (2.0 to 7.5) | 3.0 (1.0 to 8.0) | 0.55 (-2.03 to 3.13) | 0.668 | 0.55 (-1.94 to 3.04) | 0.669 |
| Depression score ≥ 8, n (%) | 4 (24) | 6 (30) | OR: 0.72 (0.15 to 3.10) | 0.659 | OR: 0.72 (0.16 to 3.13) | 0.659 |

CI: confidence interval; HADS: Hospital Anxiety and Depression Scale (high score denotes greater anxiety / depression level); OR: Odds ratio.

The HADS had a median respond time of 108.5 (IQR 98 to 121) and 371 days (IQR 362 to 388)

^a^ Continuous variables are expressed as differences (β estimate) in scores between the haloperidol and placebo group (reference), and categorical variables as OR.

^b^ Adjusted for a random effect for hospital. There were no baseline differences between the haloperidol and placebo group.

^c^ Adjusted analyses were not possible due to risk of overfitting (low number of patients/events relative to the number of covariates).

## Online Supplement 5- Fig 1. The distribution of PTSD and anxiety and depression symptoms.


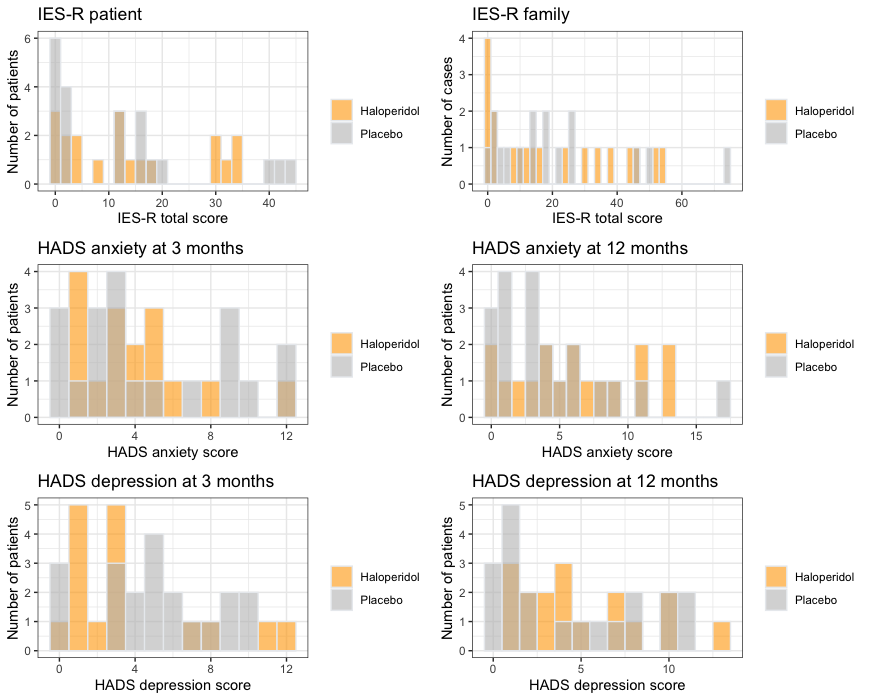


HADS: hospital anxiety and depression scale; IES-R: impact of event scaler revised.

There were no differences in IES-R total scores for patient and familky members, nor for the HADS anxiety and depression scores both at 3 and 12 months.

## Online Supplement 5 - Table 4. Haloperidol and long-term cognitive outcomes after delirium in critically ill patients, at 3 and 12 months after randomization in the ICU.

| **Cognitive domain ^a^** | **Haloperidol** | **Placebo** | **Unadjusted difference (95%CI) ^b^** | **p value** | **Adjusted difference (95%CI) ^c^** | **p value** |
| --- | --- | --- | --- | --- | --- | --- |
| *3 months after randomization (haloperidol: n = 16; placebo: n = 17)^d^* | | | | |  |  |
| MoCA score, median (IQR) | 25.0 (20.3 to 28.0) | 27.0 (25.0 to 28.0) | -1.63 (-4.51 to 1.25) | 0.256 | -1.63 (-4.39 to 1.13) | 0.256 |
| Impaired general cognition, n (%) | 8 (50) | 5 (29) | OR: 2.40 (0.59 to 10.65) | 0.231 | OR: 2.40 (0.57 to 10.04) | 0.231 |
| Memory | | | | | | |
| Relative memory capacity score, mean (SD) | 31.3 (13.4) | 33.1 (10.9) | -1.87 (-10.51 to 6.78) | 0.663 | -2.64 (-9.95 to 5.03) | 0.485 |
| Delayed recall score, median (IQR) | 4.0 (2.0 to 8.5) | 6.0 (4.0 to 8.0) | -0.58 (-3.03 to 1.87) | 0.632 | -0.59 (-2.93 to 1.77) | 0.630 |
| Recognition memory score, median (IQR) | 26.5 (21.0 to 28.0) | 28.0 (25.0 to 29.5) | -1.11 (-4.46 to 2.24) | 0.505 | -1.36 (-4.14 to 1.52) | 0.345 |
| Language | | | | | | |
| Semantic fluency test score, median (IQR) | 19.0 (14.3 to 21.8) | 23.0 (17.5 to 27.0) | -2.61 (-7.52 to 2.31) | 0.288 | -2.61 (-7.32 to 2.11) | 0.288 |
| BNT score, median (IQR) | 27.0 (24.0 to 28.0) | 27.0 (23.0 to 28.0) | 1.31 (-1.39 to 4.01) | 0.331 | 1.24 (-1.28 to 3.89) | 0.336 |
| WAIS-IV score, mean (SD) | 13.8 (3.6) | 12.6 (4.0) | 1.17 (-1.54 to 3.87) | 0.387 | 1.19 (-1.37 to 3.72) | 0.358 |
| Trail making test A score, median (IQR) | 55.5 (27.8 to 64.3) | 40.0 (24.5 to 43.5) | 6.97 (-31.05 to 44.98) | 0.711 | 6.97 (-29.50 to 43.43) | 0.711 |
| Trail making test B score, mean (IQR) | 150.0 (88.2) | 123.8 (77.4) | 26.24 (-32.58 to 85.05) | 0.37 | 26.24 (-30.18 to 82.65) | 0.370 |
| *12 months after randomization (haloperidol: n = 9; placebo: n = 11) ^d^* | | | | | | |
| MoCA score, median (IQR) | 27.0 (20.5 to 28.5) | 27.0 (25.0 to 28.0) | -0.34 (-4.14 to 3.46) | 0.852 | -0.34 (-3.88 to 3.19) | 0.852 |
| Impaired general cognition, n (%) | 3 (33) | 5 (46) | OR: 0.60 (0.09 to 3.66) | 0.583 | OR: 0.60 (0.10 to 3.72) | 0.583 |
| Memory | | | | | | |
| Relative memory capacity score, mean (SD) | 31.6 (11.5) | 35.8 (12.9) | -4.26 (-15.88 to 7.35) | 0.451 | -5.71 (-15.86 to 6.16) | 0.294 |
| Delayed recall score, mean (SD) | 5.3 (4.1) | 7.1 (4.1) | -1.76 (-5.62 to 2.10) | 0.352 | -1.76 (-5.35 to 1.83) | 0.352 |
| Recognition memory score, median (IQR) | 28.0 (23.5 to 29.5) | 29.0 (23.0 to 30.0) | -0.78 (-4.51 to 2.96) | 0.667 | -0.78 (-4.25 to 2.69) | 0.667 |
| Language | | | | | | |
| Semantic fluency test score, mean (SD) | 21.3 (8.4) | 21.0 (8.5) | 0.33 (-7.67 to 8.33) | 0.931 | 0.33 (-7.10 to 7.77) | 0.931 |
| BNT score, median (IQR) | 28.0 (21.0 to 29.0) | 28.0 (26.0 to 29.0) | -1.33 (-4.56 to 1.89) | 0.397 | -1.33 (-4.33 to 1.66) | 0.397 |
| WAIS-IV score, mean (SD) | 14.7 (3.9) | 13.5 (3.0) | 1.12 (-2.15 to 4.40) | 0.481 | 1.13 (-1.90 to 4.14) | 0.461 |
| Trail making test A score, median (IQR) | 46.0 (30.0 to 75.0) | 46.0 (22.0 to 60.0) | -0.98 (-61.46 to 59.50) | 0.973 | -0.98 (-57.18 to 55.22) | 0.973 |
| Trail making test B score, median (IQR) | 80.0 (57.0 to 231.5) | 93.0 (75.0 to 150.0) | 12.15 (-69.53 to 93.83) | 0.758 | 12.15 (-63.76 to 88.06) | 0.758 |

BNT: Boston Naming Test; CI: confidence interval; IQR: interquartile range; MoCA: Montreal Cognitive Assessment; OR: odds ratio; SD: standard deviation.

^a^ General cognition was assessed with the MoCA (maximum score is 30 [patients with education <12 years obtained an extra score of 1], with higher scores indicating a better cognition and a score <26 considered as generally impaired cognition) ^22^. Memory was assessed with the Rey Auditory Verbal Learning Test (TALVT) ^23^: relative memory capacity (total score of 5 trials, maximum score 75), delayed recall (maximum score 15) and recognition memory (maximum score 30), with higher scores reflecting better memory. Language as verbal fluency with Semantic fluency test ^24^ by total number of animals summed up in one minute (with higher scores indicating better fluency), and as confrontational naming abilities using the BNT, short version, with maximum score 30, with higher scores indicating better naming ^27^. Processing speed was assessed with the Digit Span (WAIS-IV) (maximum score is 30, higher scores indicating better processing speed) ^25^, attention with Trail making tests A (maximum score is 300 seconds, lower scores reflect better attention) ^26^, and executive functioning with the Trail making test B (maximum score 300 seconds, with lower scores reflecting better executive functioning) ^27^.

^b^ Expressed as differences, unless mentioned otherwise. The differences (β coefficient) indicate the differences in scores for patients who were randomized to haloperidol versus those randomized to placebo (reference).

^c^ Adjusted for a random effect for hospital. There were no baseline differences between the haloperidol and placebo group.

^d^ Four patients who were not tested at 3 months were tested at 12 months, and 17 patients who were tested at 3 months were not assessed at 12 months.

## Online Supplement 5 - Fig. 2 Differences in cognitive domains between the haloperidol and placebo group at 3 and 12 months after randomization


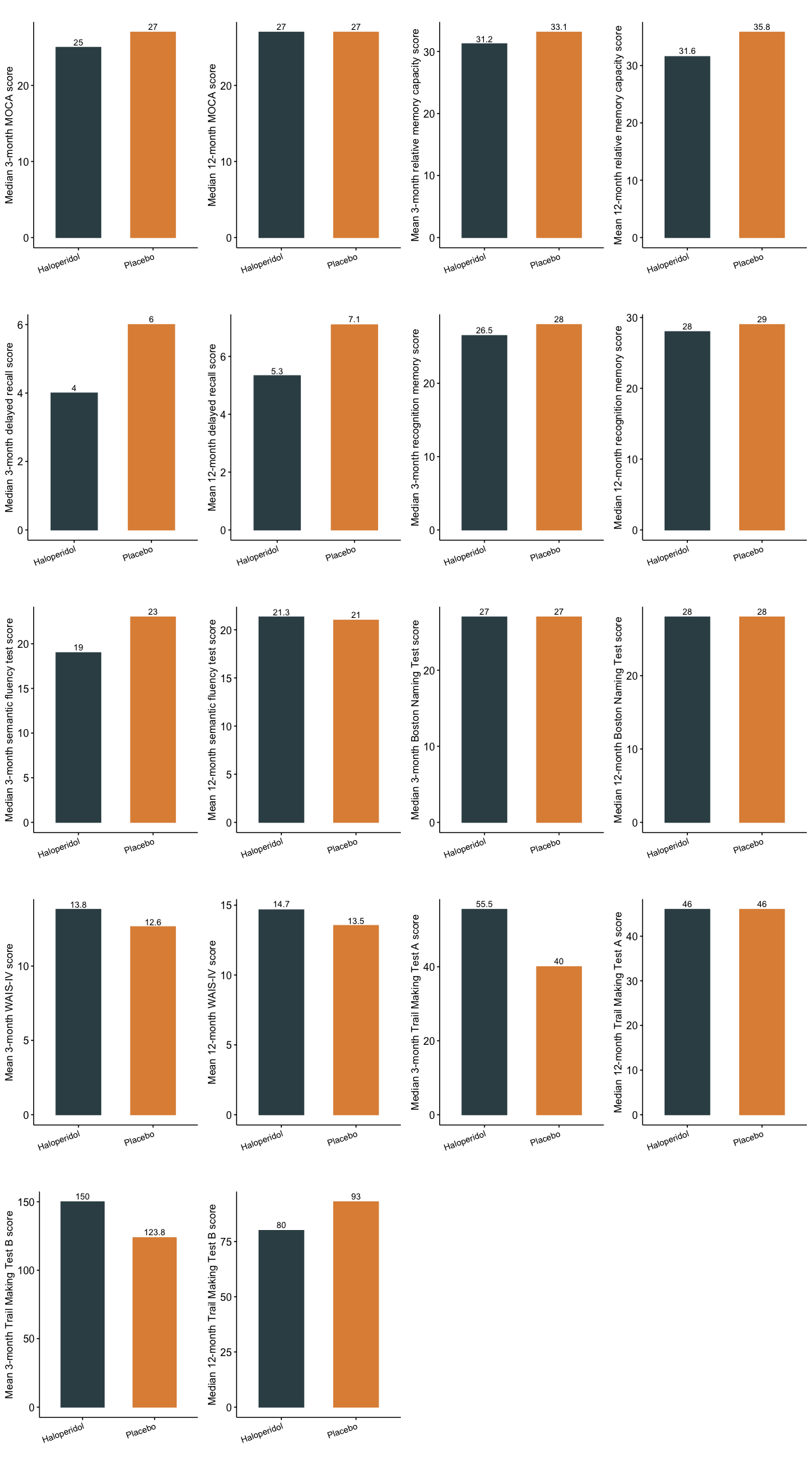


Both at 3 and 12 months after randomization, there were no differences in any of the cognitive domains

between patients treated with haloperidol and patients in the placebo group.

## Online Supplement 5 – Table 5. Difference in improvement of cognitive functioning between the haloperidol and placebo group from 3 to 12 months.

| **Cognitive domain ^a^** | **Haloperidol (n = 7)** | **Placebo (n = 9)** | **Unadjusted difference (95%CI) ^b^** | **p value** | **Adjusted difference (95%CI) ^c^** | **p value** |
| --- | --- | --- | --- | --- | --- | --- |
| MoCA score, mean (SD) | 0.3 (0.8) | 0.2 (2.4) | 0.06 (-1.96 to 2.09) | 0.947 | 0.06 (-1.77 to 1.90) | 0.947 |
| Memory | | | | | | |
| Relative memory capacity score, mean (SD) | 2.0 (4.4) | 5.3 (7.6) | -3.33 (-10.29 to 3.62) | 0.322 | -3.33 (-9.66 to 2.99) | 0.322 |
| Delayed recall score, mean (SD) | 0.0 (2.5) | 1.9 (3.5) | -1.89 (-5.37 to 1.49) | 0.251 | -1.66 (-4.96 to 1.18) | 0.308 |
| Recognition memory score, median (IQR) | 1.0 (0.0 to 3.0) | 0.0 (-3.0 to 4.5) | -0.54 (-7.50 to 6.42) | 0.870 | 1.29 (-4.24 to 5.99) | 0.605 |
| Language | | | | | | |
| Semantic fluency test score, mean (SD) | 4.0 (8.3) | 0.1 (3.2) | 3.89 (-2.55 to 10.33) | 0.216 | 3.89 (-1.96 to 9.74) | 0.216 |
| BNT score, median (IQR) | 0.0 (-5.0 to 1.0) | 1.0 (0.0 to 4.5) | -4.29 (-8.74 to 0.17) | 0.058 | -3.07 (-7.99 to 0.34) | 0.111 |
| WAIS-IV score, median (IQR) | -1.0 (-2.0 to 0.0) | 2.0 (0.5 to 3.0) | -1.92 (-3.97 to 0.13) | 0.064 | -1.95 (-3.78 to -0.06) | 0.061 |
| Trail making test A score, mean (SD) | -1.6 (19.3) | 5.4 (24.3) | -7.02 (-31.08 to 17.05) | 0.542 | -2.24 (-25.32 to 16.57) | 0.822 |
| Trail making test B score, median (IQR) | -4.0 (-9.0 to 0.0) | -20.0 (-46.5 to 1.5) | 3.13 (-61.77 to 68.02) | 0.919 | 3.13 (-55.85 to 62.10) | 0.919 |

BNT: Boston Naming Test; CI: confidence interval; IQR: interquartile range; MoCA: Montreal Cognitive Assessment; SD: standard deviation

^a^ Differences between the two assessment periods are calculated by subtracting the score at 3 months from the score of 12 months for all cognitive subdomains, in patients who underwent both neurocognitive assessments. With exception for the Trail making test A and B scores, a positive value indicates improvement of functioning on that domain, whereas a negative value indicates deterioration.General cognition was assessed with the MoCA (maximum score is 30 [patients with education <12 years obtained an extra score of 1], with higher scores indicating a better cognition and a score <26 considered as generally impaired cognition) ^22^. Memory was assessed with the Rey Auditory Verbal Learning Test (TALVT) ^23^: relative memory capacity (total score of 5 trials, maximum score 75), delayed recall (maximum score 15) and recognition memory (maximum score 30), with higher scores reflecting better memory. Language as verbal fluency with Semantic fluency test ^24^ by total number of animals summed up in one minute (with higher scores indicating better fluency), and as confrontational naming abilities using the BNT short version, with maximum score 30, with higher scores indicating better naming) ^27^. Processing speed was assessed with the Digit Span (WAIS-IV) (maximum score is 30, higher scores indicating better processing speed) ^25^, attention with Trail making tests A (maximum score is 300 seconds, lower scores reflect better attention) ^26^, and executive functioning with the Trial making test B (maximum score 300 seconds, with lower scores reflecting better executive functioning) ^27^.

^b^ The differences (β coefficient) indicate the differences in scores for patients who were randomized to haloperidol versus those randomized to placebo (reference).

^c^ Adjusted for a random effect for hospital. There were no baseline differences between the haloperidol and placebo group.

## Online Supplement 5 – Table 6. Haloperidol and long-term quality of life assessed with Short Form-36 after delirium in critically ill patients, at 3 and 12 months after randomization in the ICU.

| **Short Form-36 subdomain ^a^** | **Haloperidol** | **Placebo** | **Unadjusted difference (95%CI) ^b^** | **p value** | **Adjusted difference (95%CI) ^c^** | **p value** |
| --- | --- | --- | --- | --- | --- | --- |
| *3 months after randomization (haloperidol: n = 18; placebo: n = 24)* | | | | |  |  |
| Physical functioning, median (IQR) | 40.0 (13.8 to 90.0) | 45.0 (16.3 to 75.0) | 0.35 (-20.77 to 21.46) | 0.974 | 0.35 (-20.10 to 20.80) | 0.974 |
| Role limitations due to physical health, median (IQR) | 0.0 (0.0 to 50.0) | 0.0 (0.0 to 25.0) | 9.03 (-14.11 to 32.17) | 0.435 | 9.03 (-13.38 to 31.44) | 0.435 |
| Role limitations due to emotional problems, median (IQR) | 100.0 (0.0 to 100.0) | 16.5 (0.0 to 100.0) | 19.86 (-9.25 to 48.97) | 0.176 | 19.86 (-8.33 to 48.05) | 0.176 |
| Energy/fatigue, mean (SD) | 50.6 (23.7) | 45.7 (19.4) | 4.85 (-8.61 to 18.30) | 0.471 | 4.13 (-8.18 to 17.88) | 0.539 |
| Emotional well-being, mean (SD) | 78.4 (14.3) | 71.3 (19.4) | 7.11 (-3.89 to 18.11) | 0.199 | 7.11 (-3.54 to 17.76) | 0.199 |
| Social functioning, mean (SD) | 52.2 (34.4) | 48.7 (27.9) | 3.56 (-15.86 to 22.97) | 0.713 | 3.56 (-15.25 to 22.36) | 0.713 |
| Pain, median (IQR) | 95.0 (51.3 to 100.0) | 85.0 (60.5 to 97.5) | -0.38 (-16.14 to 15.39) | 0.962 | -0.38 (-15.65 to 14.90) | 0.962 |
| General health, mean (SD) | 61.7 (12.9) | 52.9 (12.4) | 8.75 (0.78 to 16.72) | 0.032 | 8.75 (1.03 to 16.47) | 0.032 |
| Health change, median (IQR) | 25.0 (0.0 to 81.3) | 25.0 (0.0 to 50.0) | 9.03 (-12.80 to 30.85) | 0.408 | 9.03 (-12.11 to 30.17) | 0.408 |
| *12 months after randomization (haloperidol: n = 17; placebo: n = 18)* | | | | | | |
| Physical functioning, mean (SD) | 57.4 (27.9) | 55.9 (26.1) | 1.46 (-17.10 to 20.03) | 0.873 | 2.08 (-16.39 to 19.32) | 0.817 |
| Role limitations due to physical health, median (IQR) | 0.0 (0.0 to 75.0) | 25.0 (0.0 to 100.0) | -10.78 (-39.67 to 18.10) | 0.453 | -10.78 (-38.56 to 17.00) | 0.453 |
| Role limitations due to emotional problems, median (IQR) | 100.0 (33.3 to 100.0) | 100.0 (58.5 to 100.0) | -5.25 (-33.06 to 22.57) | 0.704 | -9.05 (-32.44 to 14.73) | 0.452 |
| Energy/fatigue, mean (SD) | 58.2 (19.7) | 54.7 (22.4) | 3.51 (-11.02 to 18.05) | 0.626 | 3.51 (-10.46 to 17.49) | 0.626 |
| Emotional well-being, mean (SD) | 72.7 (19.0) | 75.1 (20.0) | -2.35 (-15.79 to 11.09) | 0.724 | -2.68 (-15.28 to 10.58) | 0.684 |
| Social functioning, median (IQR) | 63.0 (44.0 to 100.0) | 76.0 (58.0 to 88.0) | 0.91 (-19.18 to 21.00) | 0.927 | 5.47 (-16.36 to 23.04) | 0.553 |
| Pain, median (IQR) | 78.0 (50.0 to 100.0) | 89.0 (65.5 to 100.0) | -7.84 (-3.57 to 7.89) | 0.318 | -0.38 (-15.65 to 14.90) | 0.962 |
| General health, mean (SD) | 55.2 (10.9) | 53.3 (12.7) | 1.84 (-6.31 to 10.00) | 0.649 | 1.84 (-6.00 to 9.69) | 0.649 |
| Health change, median (IQR) | 100.0 (50.0 to 100.0) | 100.0 (62.5 to 100.0) | 1.47 (-17.62 to 20.56) | 0.876 | 0.786 (-14.25 to 15.66) | 0.917 |

CI: confidence interval; IQR: interquartile range; SD: standard deviation.

The median time until the questionnaires were sent back was 96 (IQR 84.5 to 110.3) and 364 days (IQR 354 to 378) after randomization, respectively.

^a^ A higher score reflects a better health (range 0 to 100).

^b^ Differences (β estimate) indicate differences in scores between the haloperidol and placebo group (reference).

^c^ Adjusted for a random effect for hospital.

## Online Supplement 5 - Fig. 3 SF-36 scores for haloperidol and placebo group at 3 (a) and 12 months (b)


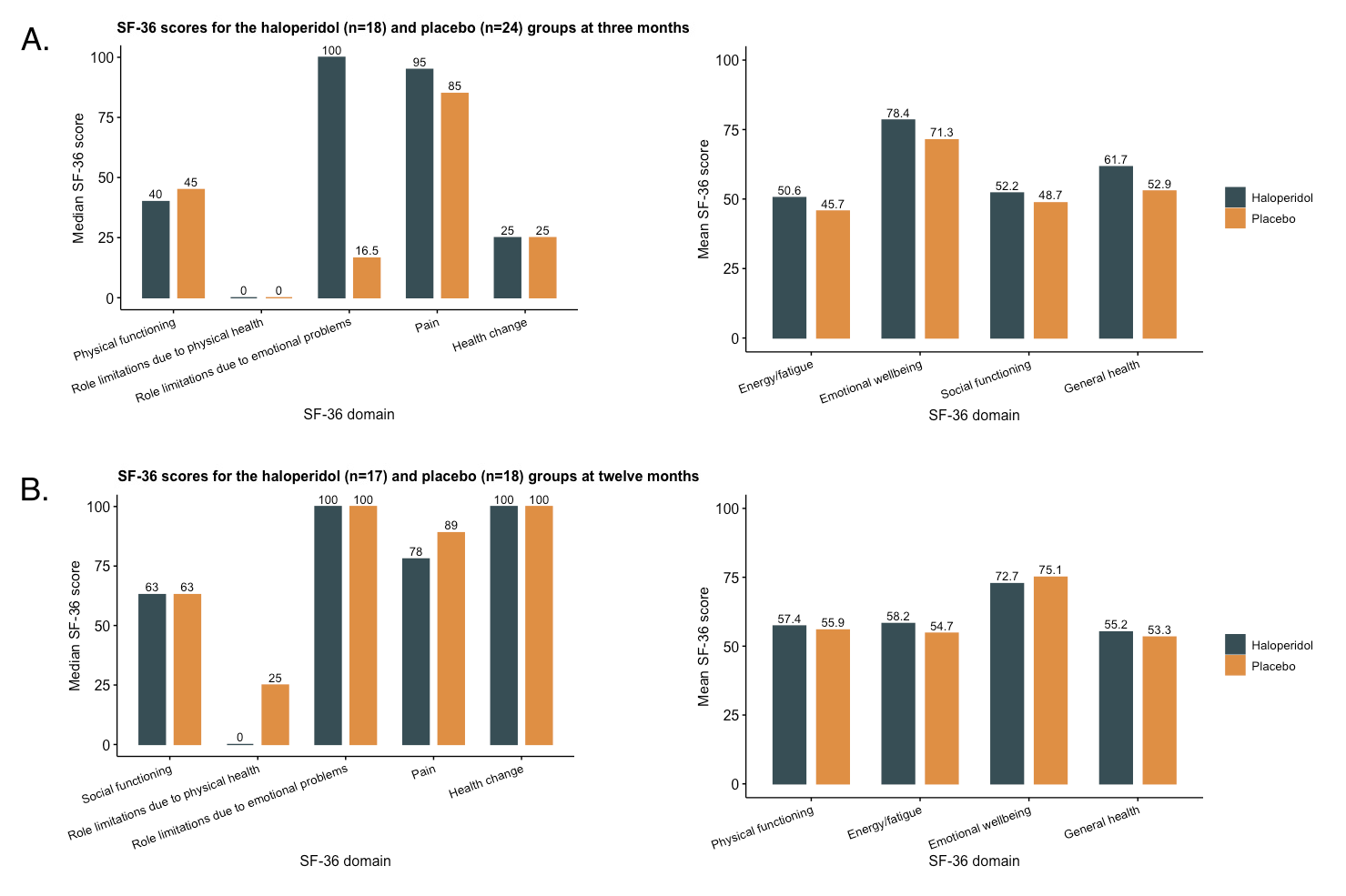


SF-36: Short-Form 36

The SF-36 consists of nine domains. The scores for each domain range from 0 to 100, with higher scores denoting better functioning on that domain. The scores are shown for each treatment group as median or mean score, depending on its distribution. Except for a significant mean difference between the haloperidol and placebo group for general health at three months, no other differences were observed.

## Online Supplement 5 - Table 7. Haloperidol and 1-year mortality after delirium.

| **Mortality** | **Haloperidol (n = 33)** | **Placebo (n = 35)** | **Unadjusted OR (95%CI)** ^a^ | **p value** | **Adjusted OR (95% CI) ^b^** | **p value** |
| --- | --- | --- | --- | --- | --- | --- |
| 1-year mortality, n (%) ^c^ | 3 (11) | 5 (17) | 0.63 (0.12 to 2.83) | 0.549 | 0.91 (0.40 to 2.06) | 0.813 |
| Time until death, days, median (95% CI) ^d^ | 321 (21.7 to 620.3) | 96 (33.7 to 158.3) | HR: 0.18 (0.02 to 1.6) | 0.122 | HR: 0.20 (0.02 to 1.77) | 0.150 |

CI: confidence interval; HR: Hazard ratio; OR: Odds Ratio.

^a^ Categorical variables are expressed as OR unless mentioned otherwise, with placebo being the reference group.

^b^ Adjusted for a random effect for hospital. There were no baseline differences between the haloperidol and placebo group.

^c^ Some values were missing: 1-year mortality 11 (16%).

^d^ Time until death was calculated as time from randomization until death, and was analysed with a mixed effects Cox proportional model and expressed as a hazard ratio (HR). Patients were censored if death had not occurred during the 1-year follow-up, if they withdrew their informed consent or were lost to follow-up during the 1-year follow-up.

# Table E5. Prespecified subgroup analyses: haloperidol versus placebo.

| **Subgroup** | **Unadjusted analyses** | | | | | **Adjusted analyses** | |
| --- | --- | --- | --- | --- | --- | --- | --- |
|  | **Haloperidol** | | **Placebo** | |  | **Adjusted RR (95%CI) ^a^** | **p value** |
|  | **n** | **No. of DCFDs, median (IQR)** | **n** | **No. of DCFDs, median (IQR)** | **p value** |  |  |
| *Delirium subtype* ^b^ | | | | | | | |
| Hypoactive delirium | 17 | 12 (5.5 to 13) | 17 | 12 (7 to 13) | 0.98 | 0.83 (0.51 to 1.34) | 0.45 |
| Mixed-type delirium | 45 | 6 (2.5 to 11) | 48 | 7.5 (2 to 10.8) | 0.92 | 0.99 (0.70 to 1.42) | 0.98 |
| *Presence of psychotic symptoms* | | | | | | | |
| No hallucinations / delusions | 10 | 11.5 (6.8 to 13) | 16 | 11.5 (0 to 12) | 0.27 | 1.07 (0.47 to 2.46) | 0.87 |
| Hallucinations / delusions | 55 | 7 (3 to 11) | 51 | 8 (3 to 11) | 0.92 | 0.97 (0.71 to 1.33) | 0.84 |
| *Delirium severity ^c^* | | | | | | | |
| Low | 22 | 6.5 (1 to 12) | 21 | 11 (8 to 12) | 0.17 | 0.92 (0.86 to 0.99) | 0.12 |
| Medium | 31 | 9 (2 to 11) | 30 | 6 (0.8 to 11) | 0.72 | 0.98 (0.60 to 1.61) | 0.93 |
| *Delirium phenotype ^d^* | | | | | | | |
| Hypoxia | 10 | 5.5 (2.8 to 10) | 8 | 11 (8.8 to 12) | 0.09 | 0.61 (0.36 to 1.04) | 0.07 |
| Sepsis | 50 | 9.5 (3 to 12) | 48 | 8 (1.3 to 11) | 0.48 | 1.05 (0.75 to 1.48) | 0.77 |
| Metabolic | 21 | 10 (3 to 11.5) | 24 | 3 (0 to 10.8) | 0.12 | 1.55 (0.86 to 2.79) | 0.15 |
| Sedation-related | 58 | 9 (3 to 12) | 60 | 8 (2 to 11) | 0.58 | 1.05 (0.76 to 1.44) | 0.76 |

CI = confidence interval; DCFD = delirium- and coma-free day; RR = relative risk

^a^ Placebo was used as a reference.

^b^ Due to low number of patients in the hyperactive delirium group (n = 3), statistical analyses were not performed.

^c^ There were no patients with a high delirium severity (mean ICDSCS score ≥7 to 8).

^d^ Patients could have multiple clinical phenotypes at randomisation^1^ hence the total count is larger than the total number of randomised patients.

# Table E6. Additional comparison of post-hoc exploratory secondary outcomes between the haloperidol and placebo group.

| **Outcome** | **Haloperidol (n = 65)** | **Placebo (n = 67)** | **Adjusted relative risk (95%CI) ^a^** | **p value** |
| --- | --- | --- | --- | --- |
| Removal of urinary catheter, no. of episodes, median (IQR) ^b^ | 1 (1 to 1.5) | 1 (1 to 2) | 0.78 (0.27 to 2.30) | 0.656 |
| Physical restraint, no. of episodes, median (IQR) ^b^ | 3 (1.3 to 4.8) | 3.5 (2 to 5) | 1 (0.73 to 1.36) | 0.997 |
| (Almost) fell or stepped out of bed, no. of episodes, median (IQR) ^b^ | 1 (1 to 2) | 1 (1 to 2) | 0.87 (0.4 to 1.91) | 0.731 |

CI = confidence interval; IQR = interquartile range; NA = not applicable; OR = odds ratio; RR = relative risk

^a^ RR unless otherwise noted. The placebo group is used as reference.

^b^ The number of episodes of an outcome were calculated only for patients who had this outcome ever during the 14-day intervention period.

# References

1. Girard TD, Thompson JL, Pandharipande PP, et al. Clinical phenotypes of delirium during critical illness and severity of subsequent long-term cognitive impairment: a prospective cohort study. *Lancet Respir Med*. 2018;6(3):213-222. doi:10.1016/S2213-2600(18)30062-6

2. Sessler CN, Gosnell MS, Grap MJ, et al. The Richmond Agitation-Sedation Scale: validity and reliability in adult intensive care unit patients. *Am J Respir Crit Care Med*. 2002;166(10):1338-1344. doi:10.1164/rccm.2107138

3. Bergeron N, Dubois MJ, Dumont M, Dial S, Skrobik Y. Intensive Care Delirium Screening Checklist: evaluation of a new screening tool. *Intensive Care Med*. 2001;27(5):859-864.

4. Ely EW, Inouye SK, Bernard GR, et al. Delirium in mechanically ventilated patients: validity and reliability of the confusion assessment method for the intensive care unit (CAM-ICU). *JAMA*. 2001;286(21):2703-2710. doi:jce10051 [pii]

5. Trogrlic Z, van der Jagt M, Lingsma H, et al. Improved Guideline Adherence and Reduced Brain Dysfunction After a Multicenter Multifaceted Implementation of ICU Delirium Guidelines in 3,930 Patients. *Crit Care Med*. 2019;doi:10.1097/CCM.0000000000003596

6. Richards KC, O'Sullivan PS, Phillips RL. Measurement of sleep in critically ill patients. *J Nurs Meas*. 2000;8(2):131-144.

7. Hodgson C, Needham D, Haines K, et al. Feasibility and inter-rater reliability of the ICU Mobility Scale. *Heart Lung*. 2014;43(1):19-24. doi:10.1016/j.hrtlng.2013.11.003

8. Vincent JL, Moreno R, Takala J, et al. The SOFA (Sepsis-related Organ Failure Assessment) score to describe organ dysfunction/failure. On behalf of the Working Group on Sepsis-Related Problems of the European Society of Intensive Care Medicine. *Intensive Care Med*. 1996;22(7):707-710. doi:10.1007/bf01709751

9. Simpson GM, Angus JW. A rating scale for extrapyramidal side effects. *Acta Psychiatr Scand Suppl*. 1970;212:11-19.

10. Barnes TR. The Barnes Akathisia Rating Scale--revisited. *J Psychopharmacol*. 2003;17(4):365-370. doi:10.1177/0269881103174013

11. Girard TD, Exline MC, Carson SS, et al. Haloperidol and Ziprasidone for Treatment of Delirium in Critical Illness. *N Engl J Med*. 2018;379(26):2506-2516.

12. Engoren M, Luther G, Fenn-Buderer N. A comparison of fentanyl, sufentanil, and remifentanil for fast-track cardiac anesthesia. *Anesth Analg*. 2001;93(4):859-864. doi:10.1097/00000539-200110000-00011

13. Knape JTA. Narcotische analgetica met aandacht voor bijzondere toedieningswegen. *Ned Tijdschr Geneeskd*. 1987;131(16):651-655.

14. VInkers CH, Tijdink JK, Luykx JJ, Vis R. Kiezen voor de juiste benzodiazepine: werkingsmechanisme en farmacokinetiek. *Ned Tijdschr Geneeskd*. 2012;156(A4900):1-7.

15. Howard P, Twycross R, Shuster J, Mihalyo M, Wilcock A. Benzodiazepines. *J Pain Symptom Manage*. 2014;47(5):955-964. doi:10.1016/j.jpainsymman.2014.03.001

16. Peterson JF, Pun BT, Dittus RS, et al. Delirium and its motoric subtypes: a study of 614 critically ill patients. *J Am Geriatr Soc*. 2006;54(3):479-484. doi:10.1111/j.1532-5415.2005.00621.x

17. Sakuramoto H, Subrina J, Unoki T, Mizutani T, Komatsu H. Severity of delirium in the ICU is associated with short term cognitive impairment. A prospective cohort study. *Intensive Crit Care Nurs*. 2015;31(4):250-257. doi:10.1016/j.iccn.2015.01.001

18. Jones C, Humphris G, Griffiths RD. Preliminary validation of the ICUM tool: a tool for assessing memory of the intensive care experience. doi: 10.3109/tcic.11.5.251.255. *Clin Intensive Care*. 2000;11(5):251-255. doi:10.3109/tcic.11.5.251.255

19. Breitbart W, Gibson C, Tremblay A. The delirium experience: delirium recall and delirium-related distress in hospitalized patients with cancer, their spouses/caregivers, and their nurses. *Psychosomatics*. 2002;43(3):183-194. doi:S0033-3182(02)70403-9 [pii]

10.1176/appi.psy.43.3.183

20. Weiss DS, Marmar CR, Wilson JP, Keane TM. Assessing psychological trauma and PTSD. *The Impact of Events Scale—Revised*. 1997;19:399-411.

21. Robinson BC. Validation of a Caregiver Strain Index. *J Gerontol*. 1983;38(3):344-348.

22. Nasreddine ZS, Phillips NA, Bedirian V, et al. The Montreal Cognitive Assessment, MoCA: a brief screening tool for mild cognitive impairment. *J Am Geriatr Soc*. 2005;53(4):695-699. doi:JGS53221 [pii]

10.1111/j.1532-5415.2005.53221.x

23. Bean J. Rey Auditory Verbal Learning Test, Rey AVLT. In: Kreutzer JS, DeLuca J, Caplan B, eds. *Encyclopedia of Clinical Neuropsychology*. Springer New York; 2011:2174-2175.

24. Ledoux K, Vannorsdall TD, Pickett EJ, Bosley LV, Gordon B, Schretlen DJ. Capturing additional information about the organization of entries in the lexicon from verbal fluency productions. *J Clin Exp Neuropsychol*. 2014;36(2):205-220. doi:10.1080/13803395.2013.878689

25. Wechsler D. Wechsler adult intelligence scale–Fourth Edition (WAIS–IV). *San Antonio, TX: NCS Pearson*. 2008;22:498.

26. Reitan RM, Wolfson D. The Halstead–Reitan neuropsychological test battery for adults—Theoretical, methodological, and validational bases. In: Grant I, Adams KM, eds. *Neuropsychological assessment of neuropsychiatric and neuromedical disorders*. 3 ed. Oxford University Press; 2009:3-24:chap 1.

27. Roth C. Boston Naming Test. In: Kreutzer JS, DeLuca J, Caplan B, eds. *Encyclopedia of Clinical Neuropsychology*. Springer New York; 2011:430-433.

28. Zigmond AS, Snaith RP. The hospital anxiety and depression scale. *Acta Psychiatr Scand*. 1983;67(6):361-370. doi:10.1111/j.1600-0447.1983.tb09716.x

29. Ware JE, Jr., Sherbourne CD. The MOS 36-item short-form health survey (SF-36). I. Conceptual framework and item selection. *Med Care*. 1992;30(6):473-483.

30. van den Boogaard M, Slooter AJC, Bruggemann RJM, et al. Effect of Haloperidol on Survival Among Critically Ill Adults With a High Risk of Delirium: The REDUCE Randomized Clinical Trial. *JAMA*. 2018;319(7):680-690. doi:10.1001/jama.2018.0160

31. Devlin JW, Skrobik Y, Gelinas C, et al. Clinical Practice Guidelines for the Prevention and Management of Pain, Agitation/Sedation, Delirium, Immobility, and Sleep Disruption in Adult Patients in the ICU. *Crit Care Med*. 2018;46(9):e825-e873. doi:10.1097/CCM.0000000000003299
